# The FACT complex associates with the RPA complex at viral replication compartments to promote adenovirus infection

**DOI:** 10.1128/jvi.00582-26

**Published:** 2026-05-26

**Authors:** Tarana Sharmin, Moxuan Zhao, Selvambigai Manivannan, Kholoud Alharbi, Kaveri Singh, Fadi S. I. Qashqari, Simon Davis, Douglas G. Ward, Andrew S. Turnell

**Affiliations:** 1 Department of Cancer and Genomic Sciences, College of Medicine and Health, The University of Birmingham 152870, Birmingham, United Kingdom; 2 Department of Metabolism and Systems Science, College of Medicine and Health, The University of Birmingham 573600, Birmingham, United Kingdom; 3 Department of Microbiology and Parasitology, College of Medicine, Umm Al-Qura University 473047, Makkah, Saudi Arabia; 4 Target Discovery Institute, Nuffield Department of Medicine, University of Oxford 105596, Oxford, United Kingdom; Tufts University School of Medicine, Boston, Massachusetts, USA

**Keywords:** adenovirus, FACT complex, RPA complex, VRCs

## Abstract

We have determined that the FACT (Facilitates Chromatin Transcription) complex components, SPT16 and SSRP1, associate with both the RPA (Replication Protein A) complex and adenovirus DNA-binding protein (DBP) within viral replication compartments (VRCs) during both human adenovirus 5 (Ad5) and adenovirus 12 (Ad12) infection, and possess proviral activities. Inactivation of FACT by the curaxin, CBL0137, reduces Ad5 and Ad12 E1A-dependent induction of early gene products, while inhibition of FACT by siRNA similarly affected Ad5 gene product synthesis, but differentially affected Ad12 gene product expression. A consistent feature of FACT inhibition, however, was a significant reduction in the protein levels of DBP, which correlated positively with a significant reduction in the formation of VRCs during infection, and the production of new, infectious virus. Pertinently, the inactivation of FACT with CBL0137 or siRNA increased the levels of the p53 protein and p53 target gene product, p21^CIP1/WAF1^; FACT inhibition by CBL0137 limited the ability of E1A to induce S-phase in Ad-infected cells. We propose, therefore, that the FACT complex cooperates with E1A to promote early gene expression and create an environment conducive to viral replication, while FACT inactivation, through its ability to modulate VRC formation and cell cycle progression, has antiviral properties.

**IMPORTANCE:**

Viruses have evolved to inhibit cellular proteins and pathways that possess inherent antiviral activity and utilize cellular proteins and pathways that possess proviral activities. Identification and characterization of both antiviral and proviral factors are therefore important toward understanding the biology of virus–host interactions and the generation of novel antivirals. Viral replication compartments (VRCs) are essential for a productive infection; they are highly dynamic, spatially organized structures to which both proviral and antiviral cellular factors are recruited. Adenoviruses recruit cellular proteins, such as the RPA complex, to VRCs, though their precise roles during infection are poorly understood. Identification and characterization of cellular, RPA complex-associated proteins at VRCs are important to understanding their multifaceted roles during infection. The studies described herein further our understanding of the relationship between adenovirus, the RPA complex, and VRCs during infection, establish proviral roles for the FACT complex, and identify FACT inhibitors as potential antivirals for adenovirus infection.

## INTRODUCTION

Human adenoviruses (Ads) have evolved to inactivate proteins and pathways that suppress viral replication and utilize host cell proteins and pathways that facilitate viral replication. As such, Ad typically inactivates proteins that are involved in the DNA damage response (DDR) and other antiviral signaling pathways through ubiquitin-targeted proteolysis, SUMOylation, gene repression, and/or chromatin regulation (1–4). For instance, Ad E1B-55K and E4orf6 proteins form a complex with either Cullin Ring Ligase (CRL) 2 or 5, dependent on Ad type, to target a large number of proteins, including p53 and MRE11, for protein degradation (5–8). E1B-55K and E4orf3 are SUMO ligases: E1B-55K SUMOylates p53 and targets it to PML nuclear tracks and targets other antiviral factors, such as the MRE11-RAD50-NBS1 (MRN) complex for SUMO-dependent degradation (9–11); while E4orf3, similarly, targets MRN to PML nuclear tracks for inactivation through SUMOylation (12). E4orf3 also has the capacity to target proteins such as TFII-I for SUMO-targeted ubiquitylation (STUbL) and proteasome-mediated degradation (13) and inhibits p53 transactivation by promoting histone H3K9 trimethylation and heterochromatin formation at p53 target promoters (14).

Ad E1A functions primarily to regulate host and viral gene expression programs and utilizes host cell factors to promote viral and cellular gene expression programs but moreover represses the expression of cellular gene products that possess antiviral activity (15, 16). The two major forms of E1A at early times of infection are the 12S and 13S gene products, which differ by conserved region 3, CR3, which is present only in the larger 13S gene product: CR1, CR2, and CR4 are common to both proteins (17). E1A function is modular, possessing molecular recognition features that form binding sites for proteins that function to regulate transcription (16). 13S E1A is required for transactivation of viral early gene promoters and transcriptional elongation, possessing two distinct domains: a zinc finger that associates with the basal transcriptional machinery and a number of cellular transcriptional regulators, as well as a C-terminal promoter-targeting region that binds to promoter-bound transcription factors (16). 12S E1A, on the other hand, generally functions to modulate cellular transcription programs during infection, through the N-terminal region, CR1, CR2, and CR4 (15–18). For instance, 12S E1A associates with pRB to relieve pRB inhibition of E2F and drive quiescent cells into S-phase (15–18). In the absence of cellular or viral oncogenes, 12S E1A, through interaction with pRB and CBP/p300, stabilizes p53 and induces p53-dependent apoptosis (19, 20).

Viral replication compartments/centers (VRCs) are membrane-less but highly spatially organized structures that lie in close proximity to PML nuclear tracks and provide a permissive environment for viral gene transcription, mRNA processing, and DNA replication, as well as virus assembly, packaging, and release (21, 22). VRC morphology, along with cellular and viral proteome composition and genome content, is highly dynamic and reflects the temporal nature and different stages of infection (23). As such, cellular and viral factors needed for these processes are recruited to VRCs in a spatially and temporally coordinated manner, while host-cell antiviral proteins can be inactivated within VRCs by protein degradation or sequestration. The Ad DBP protein, a major component of VRCs, is encoded by the E2A transcription unit and is expressed from both early and late promoters (24). DBP, with its ability to self-oligomerize and undergo multiple post-translational modifications, functions in a similarly dynamic manner during infection and is integral to VRC formation and function (25, 26). At early stages of infection, DBP is distributed diffusely throughout the nucleus, prior to forming small intranuclear foci, nascent VRCs, that develop into ring-like structures which, during the late stages of infection, coalesce to form interconnected channels throughout the nucleus that form through liquid–liquid phase separation (25, 26). DBP was originally characterized as a single-stranded (ss) DNA-binding protein but also has the capacity to bind double-stranded (ds) DNA and DNA ends (27–29), and as such, plays important roles in viral DNA synthesis initiation and elongation, as well as stabilizing the displaced ssDNA non-template strand prior to the second round of replication (25).

RPA is a cellular ssDNA-binding protein complex that was originally identified as being essential for simian virus 40 (SV40) DNA replication (30). It comprises three subunits, RPA70 (RPA1), RPA32 (RPA2), and RPA14 (RPA3), which each possess at least one oligonucleotide-binding fold important for ssDNA association (30, 31). Through its ability to bind ssDNA and numerous cellular proteins, the RPA complex is integral to cellular DNA replication, recombination, and repair pathways, where it serves to recognize uncoated ssDNA and act as a scaffold to recruit proteins involved in these processes (32). RPA associates with VRCs during infection, where it recruits cellular proteins such as E1B-AP5 (hnRNPUL1) and SMARCAL1 (33, 34). RPA2 is phosphorylated to different extents in the later stages of Ad infection by ATR in an E1B-AP5-dependent manner (33), but the precise roles of RPA during infection are not well delineated.

The Facilitates Chromatin Transcription (FACT) complex comprises two proteins: Suppressor of Ty -SPT16 and Structure-Specific Recognition Protein 1 (SSRP1) (35, 36). FACT regulates the dynamic assembly and disassembly of nucleosomes to allow for transcription, DNA replication, the resolution of DNA:RNA hybrids (R-loops), and is important in maintaining nucleosomal and genomic stability (37–39). In this regard, FACT possesses specific histone chaperone properties and can associate with histone H2A-H2B dimers to allow for their removal from or deposition on the nucleosome (37, 38). FACT associates selectively with genes being transcribed and maintains chromatin structures in these regions, as well as playing an important role in transcriptional initiation and elongation (35, 40). FACT actively remodels nucleosome histone composition on actively transcribed genes to allow for the passage of RNAPII, such that FACT depletion leads to defective promoter pausing and transcriptional elongation, as well as the premature termination of RNAPII (41). Curaxins are carbazole-based heterocyclic compounds that were identified from a chemical library for their ability to reactivate functionally inactive wild-type (wt) p53 in renal carcinoma cell lines (42). Subsequent studies determined that one of these derivatives, CBL0137, was particularly effective in activating p53 and inhibiting NF-kB, through modulation of FACT complex function (43). It has been determined that the FACT association with transcriptionally active genes diminishes following curaxin treatment, particularly for highly transcribed genes. It is thought that curaxin removes FACT from actively transcribed genes and “traps” it on chromatin at transcriptionally inactive areas of the genome, a phenomenon broadly known as chromatin trapping (43). Curaxins are currently in phase I and II clinical trials for cancer treatment (44).

Here, we have used GFP pulldowns coupled to mass spectrometry to identify cellular proteins that associate with the RPA complex during the early stages of Ad5 and Ad12 infection. In addition to identifying known RPA-binding proteins and proteins known to associate with VRCs, we have identified a number of novel proteins not previously determined to be recruited to VRCs through RPA. Among these, we have identified the FACT complex as an RPA-interacting protein at VRCs and have established that FACT possesses proviral activities to promote a productive Ad infection.

## RESULTS

### Identification of RPA complex-interacting proteins during both Ad5 and Ad12 infection

**Fig 1:**
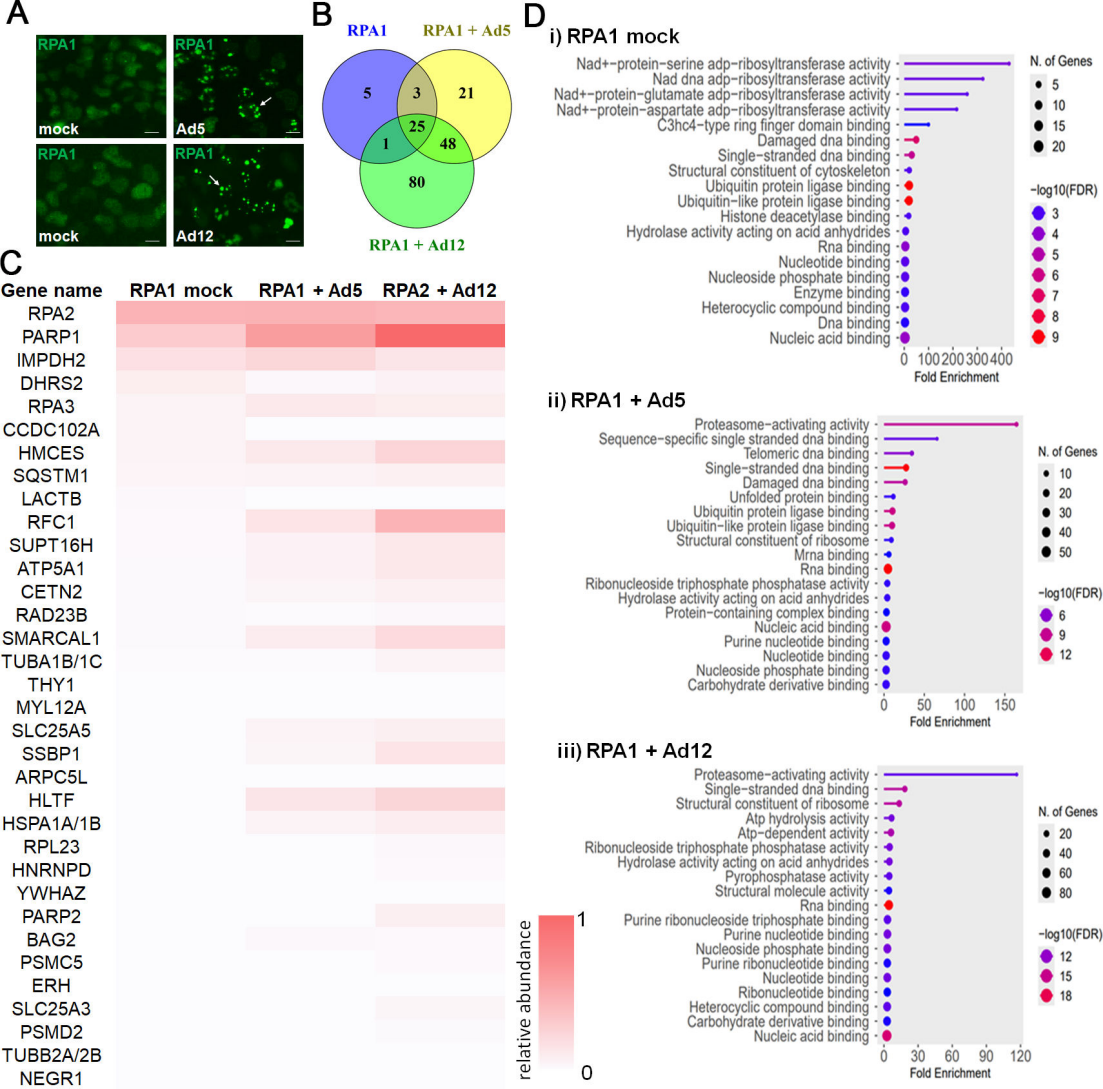
(**A**) Recruitment of GFP-RPA1 to VRCs during infection. GFP-RPA1 U2OS cells were either mock-infected or infected with Ad5 or Ad12 at 10 pfu/cell. At 24 h post-infection, images were captured with an EVOS fluorescent digital inverted microscope. Note the pan-nuclear distribution of GFP-RPA1 in mock-infected cells and GFP-RPA1 accumulation at VRCs following infection (white arrows) with either Ad5 or Ad12. Scale bar = 10 µM. (**B–D**) Mass spectrometric identification of cellular RPA1-interacting proteins following Ad5 and Ad12 infection of GFP-U2OS and GFP-RPA1-U2OS cells. At 24 h post-mock, Ad5, and Ad12 infection at 10 pfu/cell, GFP pulldowns were performed using GFP-trap beads and processed for MS. (**B**) The number and distribution of cellular RPA1-interacting proteins between mock-, Ad5-, and Ad12-infected GFP-RPA1 cells were sorted by Venn diagram using Venny 2.0.2. (**C**) Heat map shows relative abundance of proteins that are associated with GFP-RPA1 in mock-infected cells and their relative abundance following both Ad5 and Ad12 infection; relative abundance was calculated using total spectral counts for each protein identified. RPA1 was omitted from the heat map, such that RPA1-binding proteins of low abundance could be visualized. (**D**) Gene Ontology molecular function analyses were performed for all RPA1-associated proteins found by MS analyses in mock-infected, Ad5-infected, and Ad12-infected cells using ShinyGo 0.85. Fold enrichment scores and the number of proteins identified in each category are shown.

To identify RPA complex-binding proteins within VRCs during infection, we utilized GFP-U2OS and GFP-RPA1-U2OS cells that constitutively express GFP and GFP-RPA1, respectively (45). Microscopy revealed that GFP was distributed evenly between the nucleus and cytoplasm in both mock-infected and Ad5 and Ad12 infection, while GFP-RPA1, as anticipated, was distributed evenly throughout the nucleus in mock-infected U2OS cells and was reorganized to VRCs during both Ad5 and Ad12 infection (Fig. 1A). Given that GFP-RPA1 localized to VRCs following infection, akin to the endogenous RPA complex, we next subjected mock-infected and Ad5- and Ad12-infected GFP-U2OS, GFP-RPA1-U2OS, and cell lysates, at 24 h post-infection, to GFP pulldown and mass spectrometry (MS) to identify RPA1-interacting proteins. MS analysis revealed that in mock-infected, non-stressed cells, 34 distinct cellular RPA1-binding proteins were identified (Fig. 1B and C), while 97 proteins were identified following Ad5 infection (Fig. 1B; Fig. S1A), and 154 RPA1-binding proteins were identified following Ad12 infection (Fig. 1B; Fig. S1B). In total, 183 distinct proteins were identified, and their relative distribution among mock- and Ad-infected cells was determined (Fig. 1; Fig. S1); of these, a number of proteins have been validated previously as being recruited to VRCs during infection (Fig. S1).

Gene Ontology Molecular Function enrichment analysis identified proteasome-activating activities and ssDNA binding, as well as other nucleotide-binding capacities as specific functions engaged by the RPA complex following both Ad5 and Ad12 infection (Fig. 1D). FACT complex component, SPT16 (SUPT16H), was identified in high abundance as an RPA1-interacting protein following Ad infection, while its cognate-binding partner, SSRP1, was identified following Ad12 infection (Fig. S1B). Given that SPT16 and SSRP1 had not previously been characterized as being recruited to VRCs, we focused our investigation toward determining the relationship between the FACT complex and VRCs during infection.

### FACT complex components SPT16 and SSRP1 are recruited to the RPA complex during Ad infection

**Fig 2:**
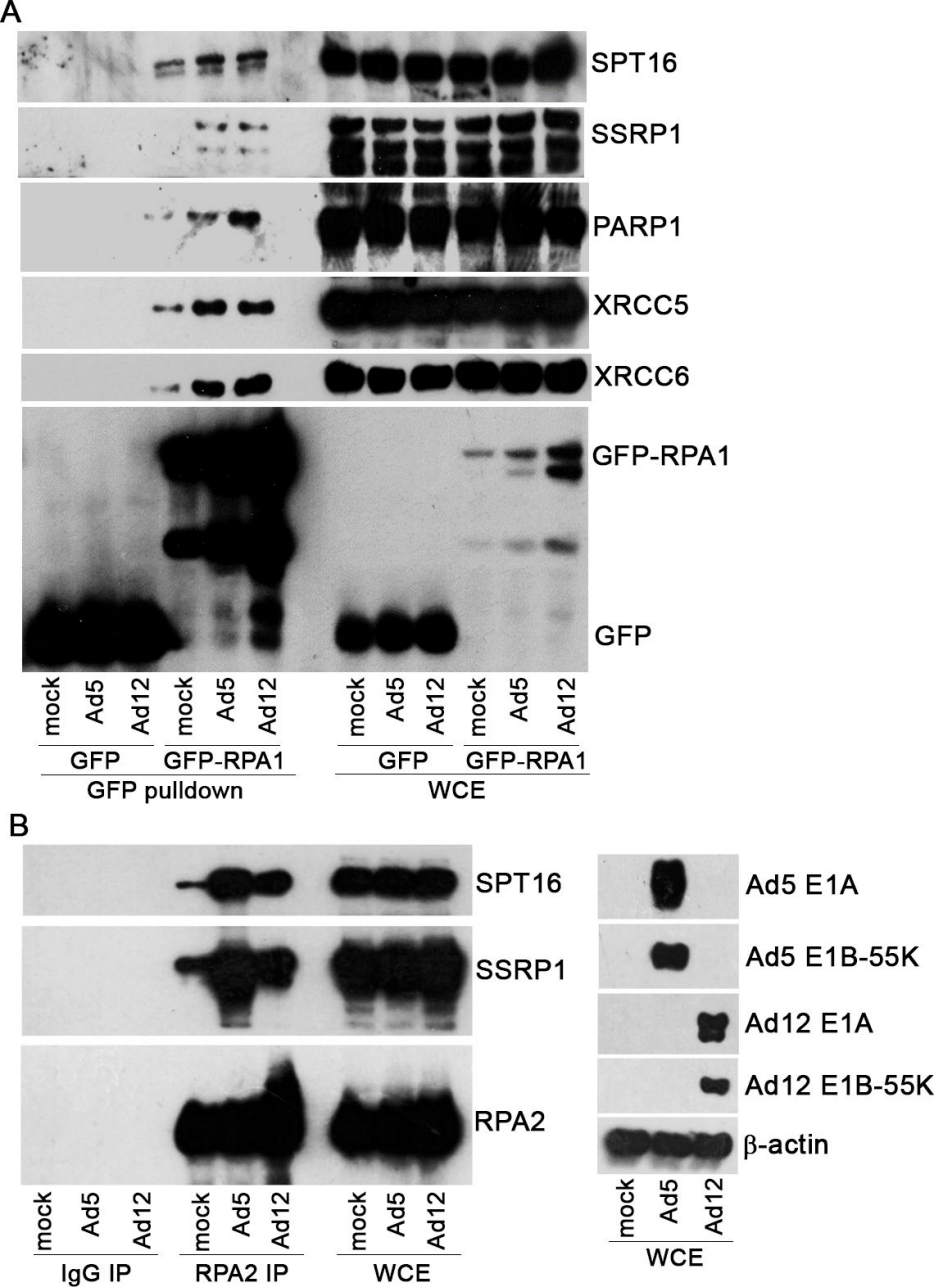
SPT16 and SSRP1 are recruited to the RPA complex during infection. (**A**) GFP U2OS and GFP-RPA1 U2OS cells were either mock-infected or infected with Ad5 or Ad12 at 10 pfu/cell. 24 h post-infection, cell lysates were subjected to GFP pulldown using GFP-trap beads. Precipitated proteins were subjected to SDS-PAGE and WB analysis with antibodies raised against FACT complex components, SPT16 and SSRP1, and RPA1-interacting proteins PARP1 and XRCC5, as well as XRCC6, to determine their interaction with RPA1 following infection. GFP and GFP-RPA1 were detected with an antibody raised against GFP. (**B**) A549 cells were either mock-infected or infected with Ad5 or Ad12 at 10 pfu/cell. 24 h post-infection, cell lysates were subjected to IP with normal rabbit IgG or anti-RPA2 antibodies and, following SDS-PAGE, subjected to WB analysis with SPT16, SSRP1, and RPA2 antibodies. Relative input levels of β-actin and Ad5 and Ad12 E1A and E1B-55K were also assessed by WB. WCE, whole-cell extract.

To validate the MS results, we performed GFP pulldowns with GFP-U2OS and GFP-RPA1-U2OS cell lines to determine whether FACT complex components, SPT16 and SSRP1, were recruited to RPA1 during Ad5 and Ad12 infection. WB analysis revealed that both SPT16 and SSRP1 were associated with RPA1 to a greater extent following both Ad5 and Ad12 infection, as did other host cell proteins identified in the MS screen and known to associate with VRCs, such as XRCC5 (and its cognate-binding partner, XRCC6) and PARP1 (Fig. 2A; 46, 47). To confirm these findings, we immunoprecipitated RPA2 from mock-, Ad5-, and Ad12-infected A549 cells and determined by WB that SPT16 and SSRP1 association with RPA2 was enhanced, relative to mock-infected cells, following both Ad5 and Ad12 infection (Fig. 2B). These data confirm that SPT16 and SSRP1 are recruited to the RPA complex during both Ad5 and Ad12 infection.

### SPT16 and SSRP1 associate with RPA and DBP at VRCs during Ad5 and Ad12 infection

**Fig 3:**
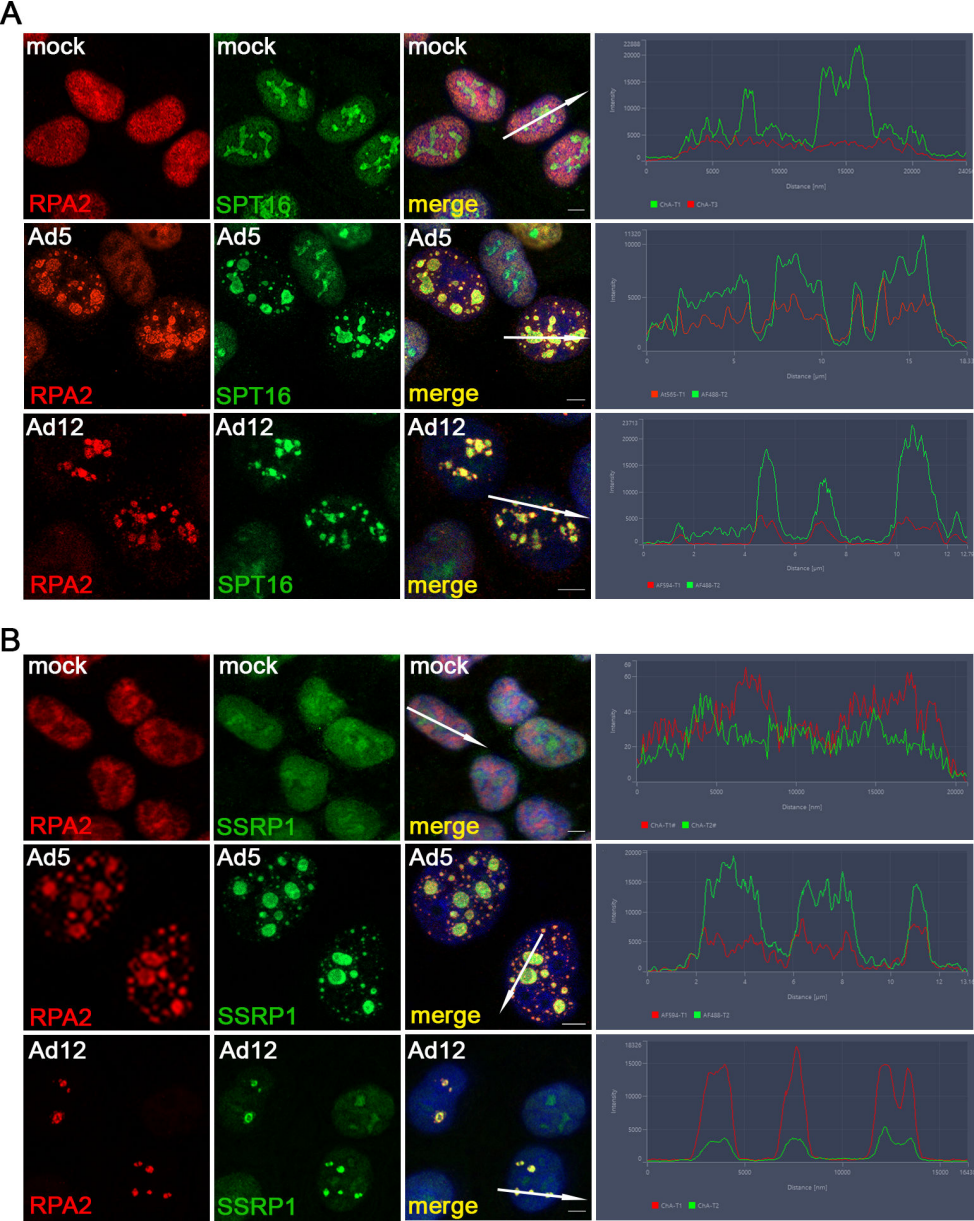
SPT16 and SSRP1 colocalize with RPA2 at VRCs in Ad5- and Ad12-infected cells. A549 cells were either mock-infected or infected with Ad5 or Ad12 at 10 pfu/cell. After 24 h, cells were fixed in methanol and co-stained with anti-RPA2 and anti-SPT16 antibodies (**A**) or co-stained with anti-RPA2 and anti-SSRP1 antibodies (**B**). Cells were counter-stained with the appropriate Alexa 488 and Alexa 594 antibodies and DAPI before visualization with a Zeiss LSM880 confocal microscope. Scale bar = 5 µM. Relative intensity plots, relating to RPA2 and SPT16, or RPA2 and SSRP1 co-staining over the arrow in the merged images, are also shown.

We next examined whether the FACT complex was recruited along with the RPA complex to VRCs following infection with Ad5 or Ad12. Confocal microscopic analysis revealed that RPA2 was distributed throughout the nucleus in mock-treated A549 cells and, consistent with other studies, was recruited to VRCs in both Ad5- and Ad12-infected A549 cells (Fig. 3). SPT16 and SSRP1 localized to nucleoli and the nucleoplasm in mock-treated cells and only partially colocalized with RPA2. However, in both Ad5- and Ad12-infected A549 cells, SPT16 (Fig. 3A) and SSRP1 (Fig. 3B) colocalized with RPA2 at VRCs; Pearson’s correlation coefficients and corresponding Costes *P* values were determined for specific regions of interest (ROIs), which indicated that there was a significant and strong positive correlation between RPA2 and SPT16/SSRP1 staining patterns following both Ad5 and Ad12 infection (Fig. S2). The 2D channel-intensity plots for specific ROIs in each image supported these findings (Fig. 3). To further these studies, we also investigated whether SPT16 and SSRP1 colocalized with Ad5 and Ad12 DBP at VRCs. Consistent with the RPA2 studies, both SPT16 and SSRP1 colocalized with DBP at VRCs, following either Ad5 or Ad12 infection (Fig. S3A and B). These data confirm that the FACT complex is recruited to VRCs during infection and suggest that any functional activities of the FACT complex retained during Ad infection are restricted to VRCs.

### The protein levels of SPT16 and SSRP1 are stable during Ad5 and Ad12 infection

**Fig 4:**
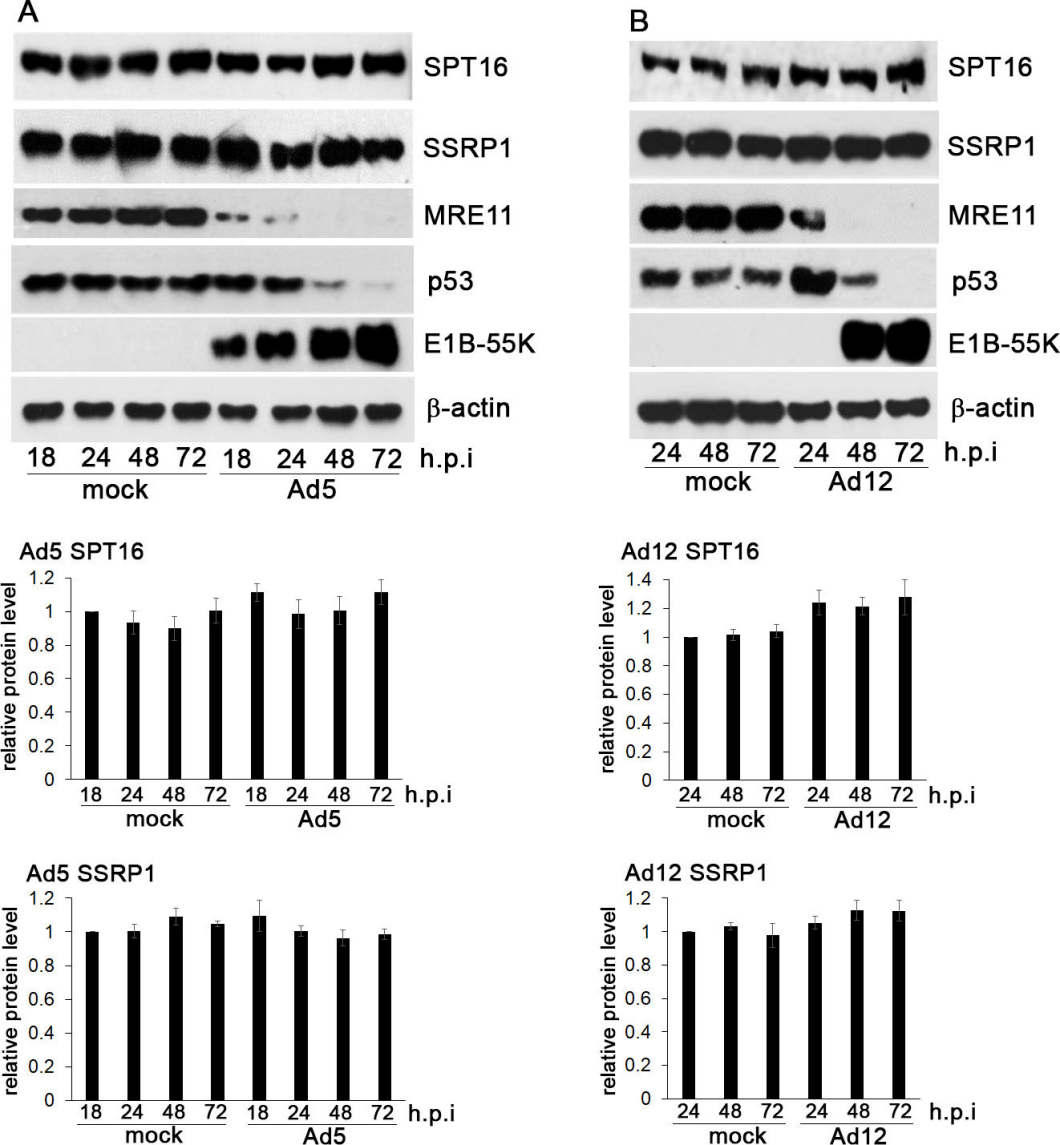
SPT16 and SSRP1 are stable during both Ad5 and Ad12 infection. A549 cells were either mock-infected or infected with Ad5 (**A**) or Ad12 (**B**) at 10 pfu/cell. Cell lysates were harvested at different time points post-infection, and samples were subjected to SDS-PAGE and WB using antibodies raised against SPT16, SSRP1, MRE11, p53, E1B-55K, and β-actin. Bar charts to indicate relative levels of SPT16 and SSRP1 during infection are shown. The relative (to β-actin) protein abundance ratio was determined by densitometric analysis with Image J (*n* = 3). Data were analyzed using a two-tailed paired t-test. Bar chart values ± S.D. indicate relative expression levels. No significant differences were seen between SPT16 and SSRP1 levels in Ad5- or Ad12-infected cells relative to mock-infected cells at any timepoint. h.p.i, hours post-infection (*P* > 0.05).

Given that cellular proteins like SMARCAL1 are degraded by the Ad-targeted CRLs following their recruitment to VRCs (34), while proteins such as E1B-AP5 are stable (33), we next wished to determine the fate of FACT complex components during Ad infection. WB revealed that SPT16 and SSRP1 were stable during both the Ad5 and Ad12 infection of A549 cells and, unlike p53 and MRE11, were not targeted for degradation (Fig. 4). These data suggest that SPT16 and SSRP1 are either inactivated by sequestration at VRCs or that they possess proviral properties that are utilized at VRCs.

### The FACT inhibitor CBL0137 attenuates Ad5 and Ad12 early region gene product expression

**Fig 5:**
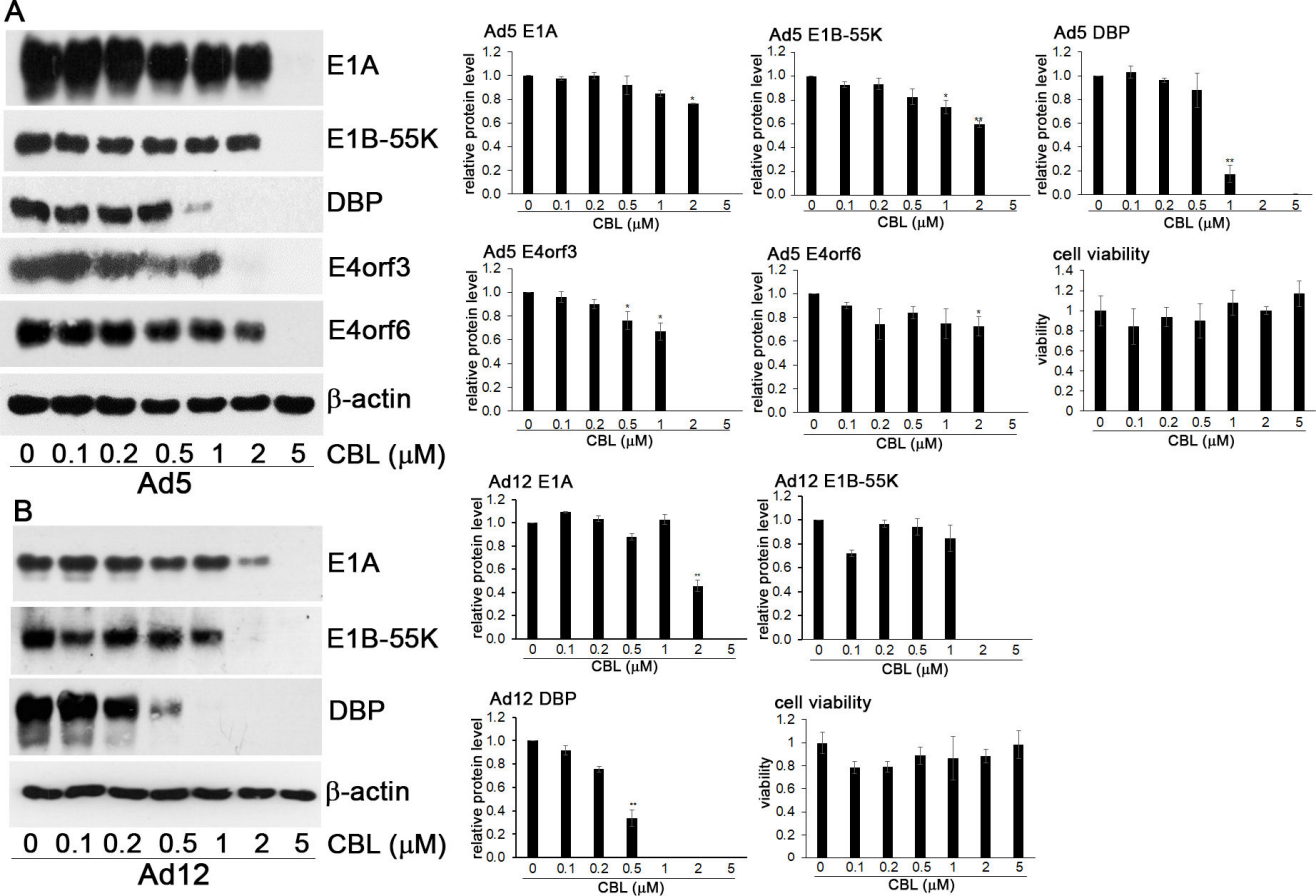
FACT inhibition with CBL0137 attenuates early gene product expression following both Ad5 and Ad12 infection. A549 cells were infected with Ad5 (**A**) or Ad12 (**B**) at 10 pfu/cell and then incubated in the absence or presence of CBL0137 (CBL). After 18 h of Ad5 infection and 24 h post-Ad12 infection, cell lysates were prepared, separated by SDS-PAGE, and analyzed by WB. For the Ad5 infection, antibodies raised against E1A, E1B-55K, DBP, E4orf3, E4orf6, and β-actin were used to assess early viral protein levels, while for the Ad12 infection, anti-E1A, E1B-55K, DBP, and β-actin antibodies were used. Bar charts to indicate relative levels of early gene products during infection are also shown. The relative (to β-actin) protein abundance ratio for early gene products was determined by densitometric analysis with ImageJ (*n* = 3). Data were analyzed using a two-tailed paired t-test. Bar chart values ± S.D. indicate the relative expression levels. *P*-values: *P ≤ 0.05, **P ≤ 0.01. Statistical differences were calculated between Ad-infected samples not treated with CBL0137 and those treated with different doses of CBL0137. The effects of CBL0137 on cell viability, relative to Ad5 and Ad12-infected cells in the absence of CBL0137, were assessed using CellTiter Glo 2.0. Data were analyzed using a two-tailed paired t-test. Bar chart values ± S.D. No significant differences were determined between Ad5- and Ad12-infected cells in the absence or presence of CBL0137 (*P* > 0.05).

Given that the FACT complex is stable during infection and localizes to VRCs, we next wished to establish the role of the complex during Ad infection. To do this, we initially determined the effects of the well-characterized FACT complex inhibitor, CBL0137, on Ad5 early region gene product expression. WB analyses revealed that treatment of Ad5-infected A549 cells with CBL0137 had a dose-dependent effect on the expression of most early gene products, which was significant at higher doses; 5 μM CBL0137 inhibited E1A expression completely, and the expression of all early gene products (Fig. 5A). It was evident from these studies that DBP expression was particularly sensitive to CBL0137 treatment and E4orf3 expression was more sensitive than E1B-55K and E4orf6; DBP levels were reduced significantly when E1A levels were also reduced between 0.5 and 2 μM but not significantly (Fig. 5A). Cell viability assays indicated that treatment of Ad5-infected cells with CBL0137, at any of the concentrations used, did not significantly affect cell viability at 18 h post-infection (Fig. 5A).

To expand upon these studies, we next investigated whether CBL0137 would similarly affect early gene product expression in Ad12-infected cells. Consistent with the Ad5 studies, WB revealed that CBL0137 had a dose-dependent effect on the protein levels of both E1B-55K and DBP (Fig. 5B). Interestingly, Ad12 E1A levels were more sensitive to FACT inhibition than Ad5 E1A, being reduced significantly at 2 μM CBL0137 and completely ablated at 5 μM (Fig. 5B). Consistent with Ad5 DBP, the levels of Ad12 DBP were reduced significantly at times when E1A levels were unaffected (Fig. 5B). Akin to the Ad5 studies, cell viability assays indicated that treatment of Ad12-infected cells with CBL0137 did not significantly affect cell viability at 24 h post-infection (Fig. 5B).

In an attempt to corroborate these findings, we determined the effect of SPT16 and SSRP1 knockdown upon the ability of Ad5 and Ad12 to initiate early gene product expression. WB analysis revealed that RNA interference reduced the levels of both SPT16 and SSRP1 significantly, although levels were only reduced by 60%–70% (Fig. S4 and S5). Consistent with the FACT inhibitor studies, dual knockdown of SPT16 and SSRP1 significantly impacted the ability of Ad5 to promote early gene product expression, although residual FACT activity in knockdown cells might mask the full requirement for FACT in early gene product expression (Fig. S4). Although FACT components were not depleted fully, Ad5 E1A expression was reduced, as were corresponding levels of E1B-55K, DBP, E4orf3, and E4orf6 (Fig. S4). FACT component knockdown also significantly impacted the ability of Ad12 to promote DBP expression (Fig. S5); Ad12 E1A and E1B-55K protein levels were not affected by FACT component knockdown, however (Fig. S5), which might relate to the remaining functional FACT activity in knockdown cells (Fig. S5).

Taken together, these data indicate that FACT inhibition affects the ability of both Ad5 and Ad12 to regulate early region gene product expression during infection and suggest that the FACT complex modulates 13S E1A-dependent early gene expression, potentially through more than one mechanism.

### Attenuation of FACT function activates the p53 pathway during Ad5 and Ad12 infection

**Fig 6:**
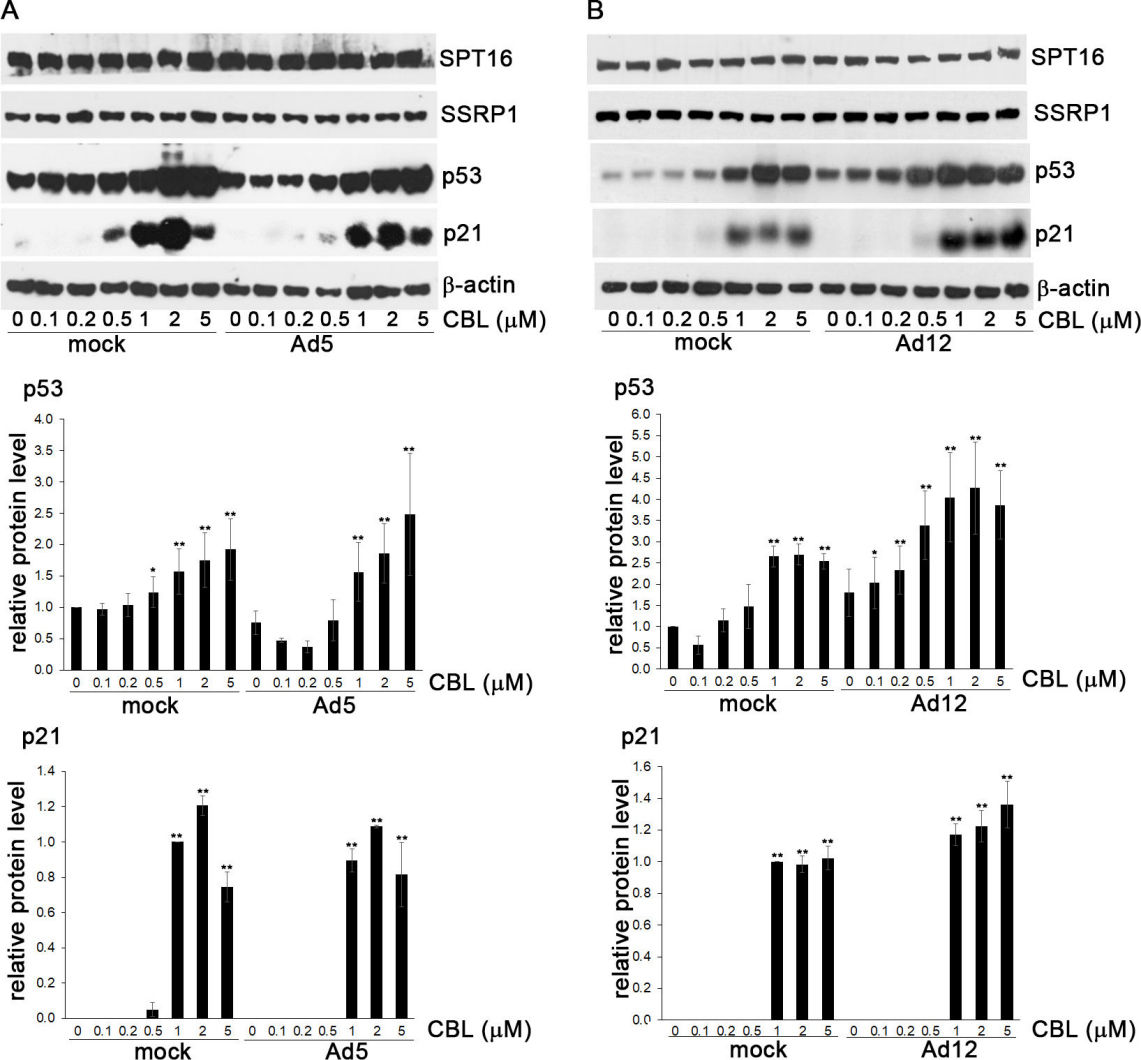
FACT inhibition with CBL0137 stabilizes p53 levels and enhances p53 transcriptional activity during Ad5 and Ad12 infection. A549 cells were either mock-infected or infected with Ad5 (**A**) or Ad12 (**B**) at 10 pfu/cell and then incubated in the absence or presence of increasing doses of CBL0137 (CBL). 24 h post-infection cell lysates were prepared, separated by SDS-PAGE, and analyzed by WB, using antibodies raised against SPT16, SSRP1, p53, p21, and β-actin. The relative (to β-actin) protein abundance ratio for p53 and p21 was determined by densitometric analysis with ImageJ (*n* = 3). Data were analyzed using a two-tailed paired t-test. Bar chart values ± S.D. indicate relative expression levels. *P*-values: *P ≤ 0.05, **P ≤ 0.01. Statistical differences were calculated between samples not treated with CBL0137 and those treated with CBL0137 for either mock-infected or Ad-infected samples.

Given that it is well established that FACT inhibition stabilizes and activates p53 (43) and that Ad targets p53 for degradation during infection (5), we next investigated whether CBL0137 stabilized and activated p53 in both mock-infected and Ad-infected cells, or whether Ad-infected cells were still able to clear p53 through targeted degradation. WB analyses revealed that CBL0137 stabilized p53 significantly and activated the p53 pathway in both mock- and Ad5-infected A549 cells, as both p53 and p21^cip1/waf1^ protein levels were elevated significantly in a CBL0137 dose-dependent manner (Fig. 6A). Akin to the Ad5 studies, the protein levels of both p53 and p21^cip1/waf1^ were also elevated significantly in both mock- and Ad12-infected A549 cells treated with CBL0137 (Fig. 6B). Consistent with these findings, siRNA-mediated knockdown of SPT16 and SSRP1 stabilized p53 significantly and activated the p53 pathway, relative to cells treated with non-silencing siRNA, in mock-infected A549 cells, as indicated by the levels of p53 and p21^cip1/waf1^ (Fig. S4 and S5). Although p21^cip1/waf1^ protein levels were reduced somewhat following both Ad5 and Ad12 infection, they were still high relative to Ad-infected cells treated with non-silencing siRNA (Fig. S4 and S5).

These data indicate that perturbation of FACT activity by both small-molecule inhibition or siRNA-mediated knockdown stabilizes p53 and promotes p53-dependent induction of p21^cip1/waf1^ in both mock-infected and Ad-infected cells.

### The FACT inhibitor CBL0137 limits the ability of Ad5 and Ad12 to induce S-phase during infection

**Fig 7:**
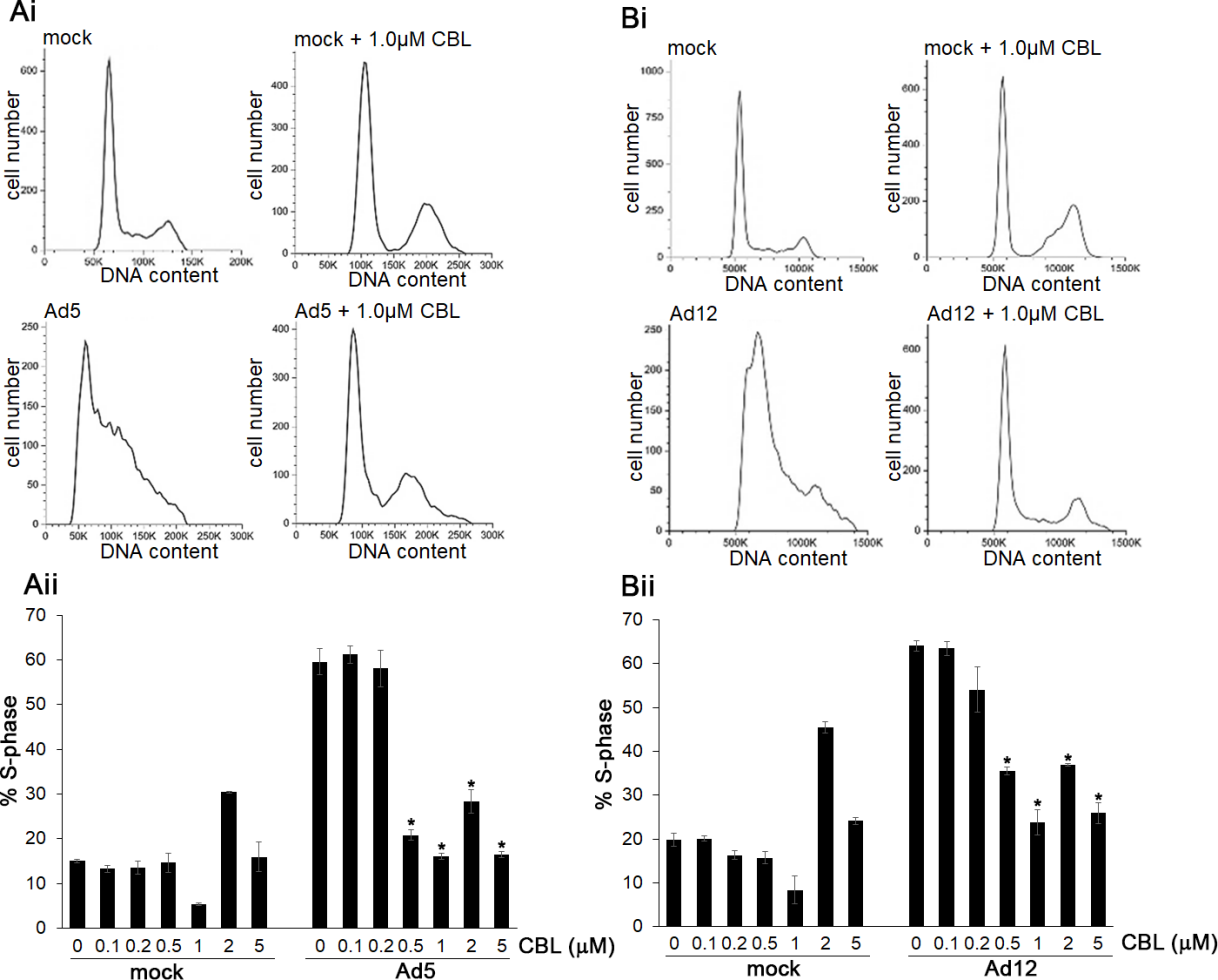
FACT inhibitor CBL0137 restricts both Ad5- and Ad12-induced S-phase entry during infection. A549 cells were mock-infected or infected with Ad5 (**A**) or Ad12 (**B**) at 10 pfu/cell and then incubated in the absence or presence of CBL0137 (CBL). At 24 h post-Ad5 infection and 32 h post-Ad12 infection, cells were fixed in 70% (vol/vol) ethanol, subjected to RNAse A treatment, resuspended in PBS containing propidium iodide, and subjected to flow cytometry analysis using a Beckman Coulter CytoFLEX S analyzer. Representative images shown in Ai and Bi were processed with Floreada.io. The relative number of cells in S-phase was determined using CytExpert software (*n* = 3). Data were analyzed using a two-tailed paired t-test. Bar chart values ± S.D. for Ad5 (Ai) and Ad12 (Bii) are shown. *P*-value: *P ≤ 0.05. Statistical differences were calculated between Ad-infected cells without CBL0137 treatment and comparable Ad-infected cells with CBL0137 treatment.

It is well established that Ad infection induces the host cell to enter S-phase through the ability of the Ad 12S E1A gene product to associate with pRB (16). In this regard, p53 growth arrest and pro-apoptotic pathways are negated during infection by its CRL-mediated degradation (5). Given that CBL0137 activates p53 transcriptionally and p21^cip1/waf1^ protein levels are elevated in both Ad5- and Ad12-infected cells (Fig. 6), it might be suspected that the ability of 12S E1A to induce S-phase entry in these cells would be affected. Cell cycle analyses revealed that in the absence of CBL0137, approximately 15% of mock-infected cells were in S-phase, 24 h post-Ad5 infection, while approximately 60% of Ad5-infected cells were in S-phase at the same time (Fig. 7A). Interestingly, CBL0137 treatment significantly affected the ability of cells to enter S-phase following Ad5 infection. At 0.5 and 1.0 μM CBL0137 treatment, the number of cells in S-phase was reduced to about 20%; this was most evident at 1 μM CBL0137 treatment (Fig. 7A). In agreement with the Ad5 studies, CBL0137 treatment also restricted the ability of Ad12-infected cells to enter S-phase, which was also most evident after treatment with 1 μM CBL0137 (Fig. 7B).

Interestingly, at 2 μM CBL0137 treatment, we observed a sub-G2/M peak in both mock- and Ad-infected cells that was coincident with where the S-phase population of cells would be seen (Fig. S6). Further examination of uninfected cells that had been treated with 2 μM and 5 μM CBL0137 revealed that, particularly at 2 μM CBL0137, approximately 20% of nuclei underwent nuclear blebbing that resulted in the generation of aneuploid cells (Fig. S6), which likely accounted for the observed sub-G2/M peak, such that cells were not genuinely in S-phase. Moreover, staining of these cells revealed that treatment of cells with 2 μM CBL0137, but more particularly 5 μM CBL0137, resulted in the formation of RPA foci, which are typically associated with sites of DNA damage and the activation of DDR pathways (48). Together, these data suggest that both Ad5 and Ad12 are unable to overcome the cell cycle block imposed by the CBL0137-mediated induction of p21^cip1/waf1^.

### The FACT inhibitor CBL0137 limits VRC formation in Ad5- and Ad12-infected cells

**Fig 8:**
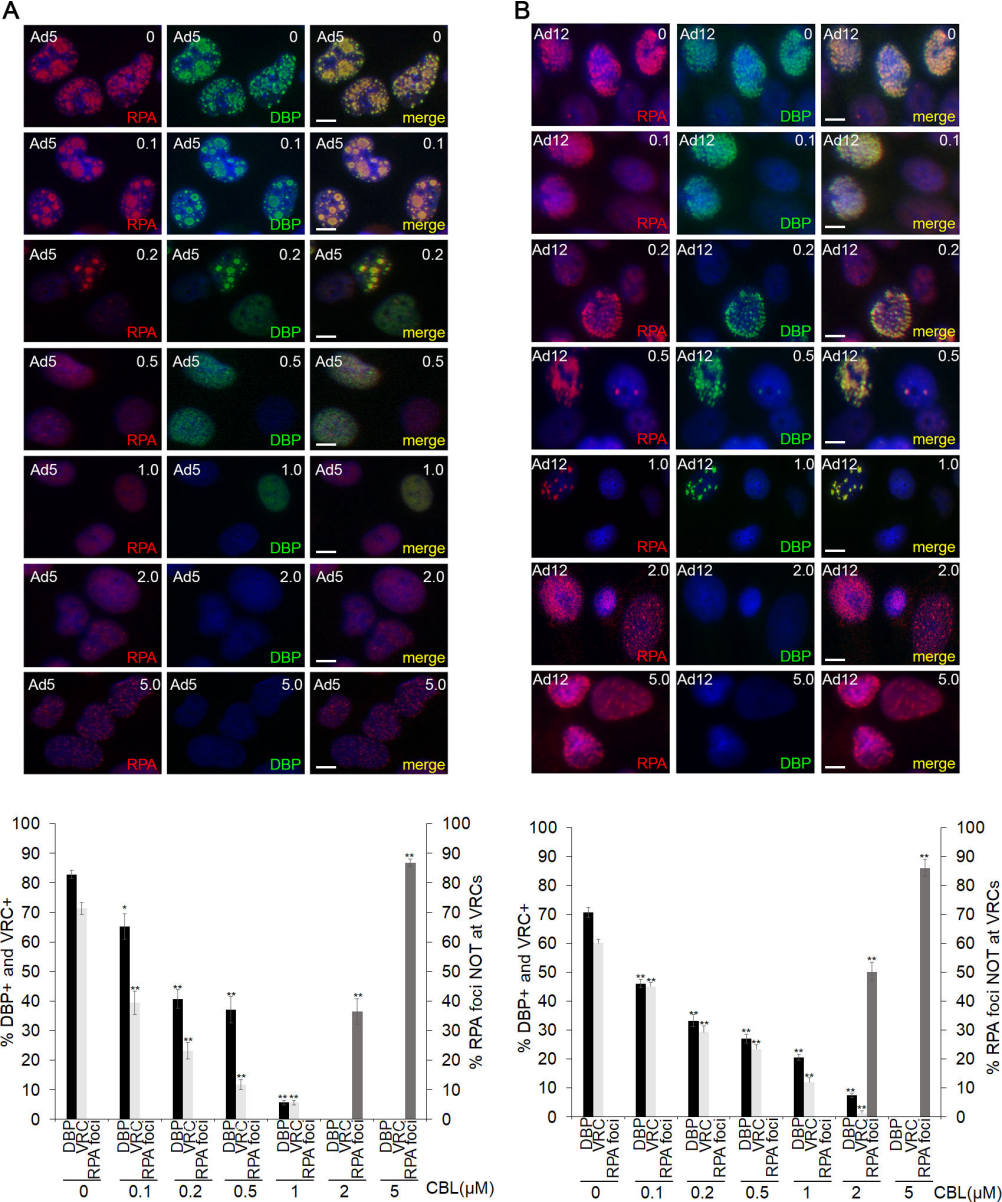
FACT inhibitor CBL0137 limits VRC formation in Ad5- and Ad12-infected cells. A549 cells were either mock-infected or infected with Ad5 (**A**) or Ad12 (**B**) at 10 pfu/cell and then incubated in the absence or presence of CBL0137 (CBL). After 24 h (Ad5) and 32 h (Ad12), infected cells were fixed in methanol and co-stained with DBP and RPA2 antibodies. Cells were then stained with appropriate Alexa 488 and Alexa 594 antibodies and DAPI before visualization with a wide-field Nikon Eclipse immunofluorescent microscope. Values in the top right of each image refer to CBL0137 concentration (μM). The number of DBP positive (DBP+), VRC+ (i.e., DBP+ and RPA2+) and purported RPA damage foci positive independent of VRCs (i.e., DBP− and RPA+) cells were counted (at least 100 cells per condition; *n* = 3). Scale bar = 5 µM. Data were analyzed using a two-tailed paired t-test. Bar chart values ± S.D. indicated the relative proportion of DBP+, VRC+, and RPA+ foci in Ad5- and Ad12-infected cells. Statistical differences were calculated for CBL0137-treated samples relative to infected cells not treated with CBL0137. *P*-values: *P ≤ 0.05, **P ≤ 0.01.

Given that DBP is important in the genesis and function of VRCs during infection and that CBL0137 attenuates DBP expression in both Ad5- and Ad12-infected cells (Fig. 5), we next wished to determine the effects of the FACT inhibitor, CBL0137, on VRC formation. At 24 h post-infection for Ad5 and 32 h post-infection for Ad12, widefield immunofluorescent microscopy revealed that, in the absence of CBL0137, mature VRCs were observed in a large proportion of infected cells, with a smaller percentage of additional DBP-positive cells where VRCs had not yet formed (Fig. 8). Interestingly, there was a significant, dose-dependent decrease in DBP-positive and VRC-positive cells, and concomitant reduction in RPA staining at VRCs, following treatment of Ad5- and Ad12-infected cells with CBL0137 (Fig. 8). It was notable that with increasing doses of CBL0137 (100 nM–500 nM of CBL0137) for Ad5 infection, there was an increased proportion of DBP-positive cells, relative to VRC-positive cells, such that VRC formation was attenuated more dramatically than DBP expression (Fig. 8A). This staining pattern was not observed as frequently for Ad12-infected cells (Fig. 8B). It was evident, however, that for both Ad5 and Ad12, there was a significant dose-dependent reduction in VRC number in response to CBL0137 treatment. Consistent with the cell cycle studies, RPA damage-like foci were observed in Ad5- and Ad12-infected cells at 2 μΜ and 5 μM CBL0137 treatment, in the absence of VRC formation (Fig. 8A and B). These data are consistent with the WB data and suggest that FACT inhibition severely impacts Ad-infected cells’ ability to synthesize DBP and form VRCs.

### The FACT inhibitor CBL0137 attenuates productive virus replication during Ad5 and Ad12 infection

**Fig 9:**
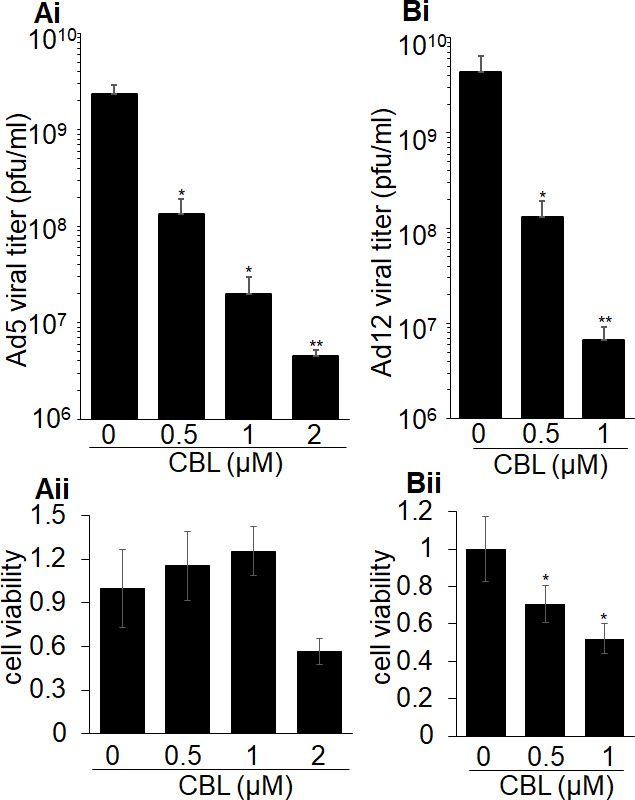
FACT inhibitor CBL0137 limits virus yield following Ad5 and Ad12 infection. A549 cells were either mock-infected or infected with Ad5 (**A**) or Ad12 (**B**) at 10 pfu/cell and then incubated in the absence or presence of CBL0137 (CBL). At 48 h post-Ad5 infection, or 72 h-post-Ad12 infection of A549 cells, cells were harvested and washed in serum-free medium before being resuspended in 1 mL of serum-free medium. Virus particles were released by four freeze-thaw cycles, with the cell debris removed by centrifugation. Virus titers were determined by plating serial diluents of cellular extract supernatants with log dilutions on either Ad5 HEK293 cells or Ad12 HER3 cells for Ad5 (Ai) and Ad12 (Bi), respectively. After infection, cells were layered with 0.75% (wt/vol) SeaPlaque agarose (Lonza) in complete DMEM. Plaques were scored after 12 days (for Ad5) and 18 days (for Ad12). Bar chart values ± S.D. *P*-values: *P ≤ 0.01, **P ≤ 0.001. Statistical differences were calculated between Ad-infected cells without CBL0137 treatment and comparable Ad-infected cells with CBL0137 treatment. The effects of CBL0137 on cell viability, relative to Ad5- and Ad12-infected cells in the absence of CBL0137, were assessed using CellTiter Glo 2.0. Data were analyzed using a two-tailed paired t-test. Bar chart values ± S.D. (*P < 0.05).

Given that CBL0137 treatment significantly reduces the levels of DBP and VRCs during infection, we next investigated whether FACT inhibition with CBL0137 also reduced the levels of productive virus produced during Ad5 and Ad12 infection. Plaque assays using cellular extracts from Ad5- and Ad12-infected cells revealed that CBL0137 attenuated significantly the yields of productive virus, relative to Ad5- and Ad12-infected cells not treated with CBL0137 (Fig. 9). As such, viral titers for Ad5 and Ad12 in the absence of FACT inhibitor were typically >10^9^ pfu/mL. Treatment of Ad5-infected A549 cells with 0.5 μM CBL0137 reduced titers approximately 15-fold, 100-fold for 1 μM CBL0137, and 400-fold for 2 μM CBL0137 (Fig. 9A). Ad12-infected cells were more sensitive than Ad5-infected cells: 0.5 μM CBL0137 reduced titers approximately 30-fold, while 1 μM CBL0137 reduced titers approximately 600-fold (Fig. 9B). Corresponding cell viability assays performed after 48 h post-infection indicated that CBL0137 did not affect cell viability of Ad5-infected cells significantly, relative to Ad5-infected cells not treated with CBL0137, although it was noted that at 2 μM CBL0137 treatment there was a reduction in cell viability, albeit not statistically significant (Fig. 9Aii). Cell viability assays conducted at 72 h post-infection resulted in a significant reduction in Ad12-infected cell viability treated with both 0.5 and 1.0 μM CBL0137 (approximately 30% and 50%, respectively), relative to Ad12-infected cells not treated with CBL0137 (Fig. 9Bii). It was clear, however, that these observed reductions in viability were not to the same level as the reductions seen in Ad12 viral titers and would not account fully for the observed reductions in viral titers (Fig. 9Bi). Taken together, these data indicate that inhibition of the FACT complex during infection and the reduction in VRCs impact significantly the number of productive virions produced in infected cells.

## DISCUSSION

To elucidate further the role of the RPA complex at VRCs during Ad infection, we performed MS analysis of GFP-RPA1 complexes isolated by GFP pulldown at the early stages of both Ad5 and Ad12 infection. This study identified a number of cellular proteins that associated with RPA1 during both Ad5 and Ad12 infection (Fig. 1; Fig. S1). Of these proteins, we investigated the role of the FACT complex, which we determined to be recruited to the RPA complex during infection (Fig. 2) and colocalize with RPA and DBP at VRCs (Fig. 3; Fig. S2 and S3). Treatment of Ad-infected cells with the FACT inhibitor, CBL0137, resulted in reduced early gene product expression, particularly DBP expression, whose protein levels did not mirror those of E1A, and which was more sensitive to inhibition than other early gene products (Fig. 5). Knockdown of FACT components, SPT16 and SSRP1, upon the ability of Ad5 and Ad12 to initiate early gene product expression was generally in agreement with the CBL0137 studies, but incomplete knockdown meant that cells retained FACT activity (Fig. S4 and S5). The reduction in DBP protein levels, following attenuation of FACT function, resulted in the dose-dependent attenuation of VRC formation and productive virus yield (Fig. 8 and 9).

The data presented here suggest that the FACT complex cooperates with 13S E1A in transactivation and transcriptional elongation of viral early genes during infection, given both their roles in initiation and elongation (36, 37, 40). However, given the differential sensitivity of early gene products, particularly DBP, to CBL0137 inhibition (Fig. 5 and 8), it is likely that the FACT complex has additional roles during infection, beyond those of cooperating directly with 13S E1A. The E2e promoter is responsible for producing DBP early during infection and, akin to other early genes, is transactivated by 13S E1A. The E2e promoter possesses E2F and CREB/ATF TF-binding sites (49, 50), such that 12S E1A can also transactivate the E2e promoter through binding to CBP/p300 (51). The E1A enhancer is also regulated by E2F, but E1B, E3, and E4 promoters, as well as the major late promoter, are not regulated by E2F (50). These observations might implicate the perturbation in E2F function following treatment with FACT inhibitor as affecting DBP expression more dramatically than other early gene products.

Consistent with this notion, we determined that the inhibition of FACT with CBL0137 or siRNA resulted in the p53-dependent activation of p21^CIP1/WAF1^ (Fig. 6; Fig. S4 and S5) and a consequent G1 arrest phenotype in both mock- and Ad-infected cells (Fig. 7). Pertinent to our study, p21^CIP1/WAF1^ is known to inhibit E2F-responsive promoters, but not E2F-unresponsive promoters, by disrupting specifically the binding of E2F-containing complexes to DNA (52). These observations could explain the more pronounced effects of CBL0137 on DBP levels in Ad-infected cells, such that p21^CIP1/WAF1^ association with E2F perturbs E2F activation of the E2e promoter. In this scenario, specific perturbation of FACT functional activity by either CBL0137 or siRNA-mediated knockdown (Fig. 6; Fig. S4 and S5) leads to the activation of the p53 pathway, which affects DBP expression. Moreover, our studies determined that at higher doses of CBL0137 and in the absence of VRC formation, distinct RPA immunofluorescent foci resembling RPA DNA damage foci (48) were observed in both mock-infected cells and in Ad-infected cells (Fig. 8; Fig. S6). It is clear, however, that more studies are needed to dissect fully the relationship between FACT inhibition and DNA damage response pathways regulated by the RPA complex. In support of the idea that CBL0137 treatment promotes genomic instability, however, we observed nuclear perturbations following treatment of both mock- and Ad-infected cells with 2 μM CBL0137. These perturbations were characterized by a sub-G2/M peak coincident with S-phase cells, which, upon examination of chromatin by IF, revealed a nuclear blebbing phenotype that resulted in aneuploidy, which might explain the cell cycle profiles observed at this particular dose of CBL0137 (Fig. S6).

The role of RPA in DNA repair, replication, and recombination is well established. RPA does, however, also participate in transcriptional regulation. For instance, it associates with the histone chaperone, HIRA, to facilitate histone H3.3 deposition at promoter and enhancer regulatory sites (53). Pertinent to the study presented here, RPA interacts directly and functionally with the FACT complex; RPA, when associated with the TF, HSF-1, recruits the FACT complex to the HSP70 promoter, to facilitate opening of the chromatin structure to allow HSF-1 access to nucleosomal DNA and promotes HSF-1-mediated gene expression (54). Additionally, studies in *S. cerevisiae* indicate that RPA localizes with transcribed regions of genes and interacts with the nontemplate strand of RNAPII complexes during elongation (55). It is plausible, therefore, that FACT cooperates with RPA during infection to promote transcriptional initiation and elongation of Ad early genes.

During the course of our investigation, the protein composition of Ad VRCs was determined (23). SSRP1 was identified and validated as being recruited to VRCs; however, the role of the FACT complex in Ad replication was not considered (23). Other viruses have been shown to employ the FACT complex during replication. The HSV-1 protein ICP22 interacts directly with the FACT complex and recruits FACT to the HSV-1 genome to promote viral gene transcription, such that an ICP22 mutant virus was defective in promoting RNAPII-dependent transcriptional elongation of HSV-1 genes (56). Follow-up studies indicated that the FACT inhibitor, CBL0137, reduced the levels of HSV-1 mRNAs and demonstrated antiviral properties by diminishing the virulence of HSV-1 in mice (57). Moreover, a related curaxin, CBL0100, acts as a potential anti-retroviral agent by inhibiting HIV-1 replication and reactivation, similarly by attenuating transcriptional elongation of viral genes (58). It has recently been determined that when the FACT complex component, SPT16, is acetylated by TIP60, it interacts with BRD4 to silence gene expression (59). Consistent with the known ability of BRD4 in stimulating interferon signaling, CBL0137 was shown to increase interferon levels and interferon-stimulated gene (ISG) expression and limit SARS-CoV-2, Zika, and influenza virus replication, demonstrating CBL0137’s potent antiviral activity (59). Given the findings detailed herein, it is clear that perturbation of FACT activity by CBL0137 and FACT-targeting siRNAs also has antiviral activity for the divergent human adenoviruses, Ad5 and Ad12. Given the number of RPA-interacting proteins identified at VRCs during Ad infection, future studies in our laboratory will focus on delineating the biological role of these proteins during infection.

## MATERIALS AND METHODS

### Cells, viruses, and drug treatment

A549, human lung carcinoma cells, Ad5 E1-transformed Human Embryonic Kidney (HEK) 293 cells, and Ad12 E1-transformed human embryonic retinal (HER) 3 cells were grown in HEPES-modified Dulbecco’s modified Eagle’s medium (DMEM; Sigma-Aldrich) supplemented with 8% (vol/vol) fetal calf serum (FCS; Sigma-Aldrich) and 2 mM L-glutamine (Sigma-Aldrich)—complete DMEM. Clonal GFP-U2OS and GFP-RPA1-U2OS cells, originally isolated from U2OS cells transfected with either pEGFPC2 alone or pEGFPC2-RPA1 (45), were maintained in complete DMEM in the presence of 200 μg/mL G418 (Gibco). All cells were maintained at 37°C in a humidified 5% CO_2_ atmosphere. wt Ad5 and wt Ad12 Huie viruses were from the ATCC, which were propagated in Ad5 HEK293 and Ad12 HER3 cells, respectively. For infection, viruses were diluted in serum-free DMEM, and cells were typically infected at a multiplicity of infection (moi) of 10 pfu/cell, unless otherwise stated. Infected cells were incubated at 37°C with agitation every 10 min. After 2-h infection, the virus-containing medium was removed and replaced with complete DMEM. The FACT inhibitor, Curaxin CBL0137 (Selleck Chemicals), was made at 10 mM stock concentrations in dH_2_O and stored at −70°C. Prior to use, CBL0137 was diluted to the appropriate working concentration (0.1 μM–5.0 μM) in complete DMEM, filtered through a 0.2 μM filter, and added to cells immediately after the 2-h viral adsorption period. Cells were typically incubated with CBL0137 for the duration of the experiment.

### Cell viability

For cell viability assays, CellTiter Glo 2.0, which quantifies cellular ATP levels as a measure of cell viability, was added to cells seeded in opaque-walled multiwell plates at the appropriate time post-infection, according to the manufacturer’s instructions (Promega). After mixing to induce cell lysis and incubation for an additional 10 min at RT to stabilize the luminescent signal, luminescence was quantified using an Envision multimode plate reader (Perkin Elmer).

### siRNA knockdown

To knock down endogenous SSRP1 and SPT16 silencer, select small-interfering RNA (siRNA) oligonucleotides (SPT16 ID- s22115; SSRP1 ID- s13491) and a non-silencing siRNA targeted against luciferase (CGUACGCGGAAUACUUCGAdTdT) were transfected using RNAiMax in Opti-MEM at a final concentration of 50 nM, and incubated at 37°C in a humidified atmosphere containing 5% CO_2_, for 6 h (all reagents from Thermo Fisher). After transfection, cells were incubated in fresh medium at 37°C in 5% (vol/vol) CO_2_, and Ad infection was performed 48 h post-RNAi treatment.

### Virus plaque assay

At appropriate times post-infection, with either wt Ad5 or wt Ad12, cells were harvested, resuspended in 1 mL of serum-free medium, and lysed by four freeze–thaw cycles, with the cell debris removed by centrifugation. Virus titers were determined by plating serial diluents of cellular extract supernatants with log dilutions on either Ad5 HEK293 cells or Ad12 HER3 cells for Ad5 and Ad12, respectively. After infection, cells were layered with 0.75% (wt/vol) SeaPlaque agarose (Lonza) in complete DMEM and overlaid with fresh agarose/DMEM every 4 days. Plaques were scored after 12 days (for Ad5) and 18 days (for Ad12).

### Cell cycle analysis by FACS

Floating cells were removed from the culture dish and retained. Following trypsinization, detached cells were added to the floating cells and subjected to centrifugation, after which the cell pellet was resuspended in 0.6 mL PBS, to which 1.4 mL ice-cold, absolute ethanol was added gently by mixing. Cells were kept at −20°C for at least 24 h. Prior to analysis, cells were pelleted by centrifugation and washed twice with PBS. Cells were then incubated in propidium iodide (10 μg/mL; Sigma Aldrich) and RNAse A (10 μg/mL; Thermo Fisher) in PBS for 1 h at 37°C. Samples were analyzed using a Beckman Coulter CytoFLEX S analyzer. Cells were gated to remove cell doublets to allow for single-cell analysis, including cells > 4N and the sub-G1 region. Data processing was performed using CytExpert software.

### Microscopy

A549 cells were plated using the Nunc Lab-Tek II Chamber Slide System (Fisher). Following the appropriate treatment, the medium was removed and cells fixed and permeabilized in methanol stored at −20°C and incubated at −20°C for 10 min. Cells were then washed three times with PBS at room temperature (RT), after which cells were incubated in 3% (vol/vol) FCS in PBS at RT for 1 h to inhibit non-specific binding. Cells were then incubated at RT for 2 h with primary antibody (diluted in 1% [vol/vol] FCS in PBS). After incubation, the cells were washed three times with PBS for 15 min at RT. Cells were then incubated in the appropriate species-specific Alexa Fluor 488 and Alexa Fluor 594 secondary antibodies (diluted in 1% [vol/vol] FCS in PBS) and stored in the dark at RT for at least 1.5 h. After this time, cells were washed three times for 15 min in PBS, and slides were mounted in ProLong Gold Antifade Mountant with DAPI (Fisher) with a glass coverslip. Cells were imaged using a Zeiss LSM-880 or LSM-900 confocal laser scanning microscope (Carl Zeiss), typically using a 63× oil immersion objective, with each channel (laser) at appropriate wavelengths configured to cover an identical optical slice. The resulting images were processed using a Zeiss LSM image browser, and captured images were edited using Adobe Photoshop or ImageJ. The colocalization plug-in tool in Fiji/ImageJ (Coloc2) was used to analyze statistically, colocalization between FACT components and RPA2, and FACT components and DBP. Alternatively, a Nikon Y-FL epi-fluorescence microscope was used to capture widefield images, which were processed using Adobe Photoshop.

### SDS-PAGE and Western blot analysis

Whole-cell protein lysates were prepared in 9M urea, 150 mM β-mercaptoethanol, 50 mM Tris-HCl (pH 7.4), and clarified by sonication and centrifugation. Following determination of protein concentrations by Bradford assay (Bio-Rad), proteins were separated by SDS-PAGE in the presence of 100 mM Tris, 100 mM Bicine, and 0.1% (wt/vol) SDS. Proteins were then transferred onto nitrocellulose membranes (Amersham) in transfer buffer (50 mM Tris, 190 mM glycine, 20% [vol/vol] methanol), and then blocked with 5% (wt/vol) dried milk powder in TBST (Tris-buffered saline containing 0.1% [vol/vol] Tween-80). Membranes were then incubated overnight with primary antibodies at the appropriate dilution in TBST containing 5% (vol/vol) milk at 4°C with agitation, after which membranes were washed four times in TBST and then incubated with the appropriate HRP-conjugated secondary antibody in TBST containing 5% (vol/vol) milk at RT for 3 h with agitation. Finally, membranes were washed four times in TBST, and antigens were detected using enhanced chemiluminescence (ECL) reagents (Millipore) and autoradiography film (SLS), using an X-ograph Compact X4 developer. Densitometric analysis to quantify protein bands from WB films was performed using ImageJ (60).

### Antibodies

The Ad5 E1A mouse monoclonal antibody (mAb), M58, Ad12 E1A mAb, #13, Ad5 E1B-55K mAb, 2A6, Ad12 E1B-55K mAb, XPH9, and the p53 mAb, DO-1 were purified from hybridoma cell lines. The Ad5 DBP mAb (B6-8) and β-actin mAb (AC-74) were from Merck. The Ad5 E4orf3 (6A11) and E4orf6 (RSA3) mAbs were a gift from Thomas Dobner. The MRE11 Ab (ab154480) was from Abcam. The Ad12 DBP sheep polyclonal antibody was made in conjunction with the MRCPPU (Dundee, U.K.). SPT16 and SSRP1 antibodies were from Santa Cruz (H300; D-7), CST (D712K; E1Y8D), and Proteintech (1E4C4; 3D3H4), respectively. RPA32 antibodies were from Abcam (EPR2877Y) and CST (E8 X 5P). Normal rabbit IgG (12-370) was from Merck. Horseradish peroxidase (HRP)-conjugated secondary anti-mouse (PO44701-2), anti-rabbit (PO39901-2), antibodies (Dako) used for WB were from Agilent, and the anti-sheep HRP (31480) secondary was from ThermoFisher. The anti-mouse Alexa Fluor 488 (A-11029), anti-mouse Alexa Fluor 594 (A-11032), anti-rabbit Alexa Fluor 488 (A-21206), anti-rabbit Alexa Fluor 594 (A-21207), and anti-sheep Alexa Fluor 488 (A-11015) were from Thermofisher.

### GFP pull down and IP

Following infection, GFP-U2OS and GFP-RPA1-U2OS cells were washed twice with cold PBS and solubilized in NETN (50 mM Tris [pH 8.0], 1 mM EDTA [pH 8.0], 1% [vol/vol] IGEPAL CA-630, and 150 mM NaCl), then sonicated and clarified by centrifugation. After protein concentration determination by Bradford assay, cell lysates were subject to GFP pulldown using 30 µL GFP-sepharose beads (GFP Trap, ChromoTek) and incubated for 3 h at 4°C with rotation. GFP Trap beads were then washed five times in NETN containing 250 mM NaCl. The beads were then re-suspended in NuPAGE LDS sample buffer (Thermofisher), boiled for 5 min at 95°C, and separated by SDS-PAGE using pre-cast NUPAGE 4-12% tris/bis-tris gels (Thermofisher) to minimize contamination. Gels were run using MOPS running buffer (50 mM MOPS, 50 mM Tris, 0.1% (wt/vol) SDS, 1 mM EDTA, pH 7.7) at 100 V until fully resolved prior to processing for mass spectrometry. For IP, samples were solubilized in NETN, clarified by centrifugation after sonication, and quantified by Bradford assay. Anti-RPA2 polyclonal antibody or normal rabbit IgG was added to cell lysates and mixed overnight on a rotator at 4°C. An amount of 30 μL packed Protein G-Agaraose beads (Pierce) was then added to each sample and incubated for an additional 3 h on a rotator at 4°C. Beads were then washed five times in NETN containing 250 mM NaCl. The beads were then re-suspended in SDS sample buffer, boiled for 5 min at 95°C, and separated by SDS-PAGE prior to analysis by WB.

### Mass spectrometry and bioinformatics

After fixing gels in 50% (vol/vol) methanol and 10% (vol/vol) acetic acid, gels were rinsed in distilled water and gel slices, covering each sample lane, excised, and washed twice, by agitation, with a solution containing 50 mM ammonium bicarbonate and 50% (vol/vol) acetonitrile for 45 min at 37°C. Proteins were then reduced by incubation for 1 h at 56°C in a solution containing 50 mM dithiothreitol and 50 mM ammonium bicarbonate in 10% (vol/vol) acetonitrile. Proteins were then alkylated in 200 mM iodoacetamide, 50 mM ammonium bicarbonate, and 10% (vol/vol) acetonitrile and incubated for 30 min at RT in the dark. The protein bands were then washed three times for 15 min each at RT in 10% (vol/vol) acetonitrile/40 mM ammonium bicarbonate on a shaker and then dried in a vacuum centrifuge. Samples were then rehydrated in sequence grade modified trypsin (Promega). An equal volume of 10% (vol/vol) acetonitrile/40 mM ammonium bicarbonate was then added to the protein bands and left to incubate with agitation overnight at 37°C.

The resultant peptides were then analyzed by LC-MS/MS using a Dionex Ultimate 3000 coupled to a Bruker maXis Impact mass spectrometer. A 75 µm × 250 mm C18 Pepmap column with 3 µm particles was used at a flow rate of 350 nL/min. Peptides were separated with a 35-min linear gradient from 1% to 40% (vol/vol) acetonitrile with 0.1% (vol/vol) formic acid. The mass spectrometer was operated in data-dependent mode, allowing for up to 5 MS/MS per cycle at an acquisition rate of 1 Hz.

Raw data files were searched against a concatenated Human, Ad5, Ad12, and common contaminant UniProt database (retrieved 18/01/2017, 93014 entries) with the Andromeda search engine (61) inside MaxQuant version 1.5.7.4 (62). Methionine oxidation and protein N-terminal acetylation were allowed as variable modifications, and cysteine carbamidomethylation was defined as a fixed modification. Peptide and protein false discovery rates (FDR) were set to 1% and determined by searching a reversed database. Enzyme specificity was set to trypsin; up to two missed cleavages and a minimum peptide length of 7 were allowed. Peptide precursor and fragment mass tolerances were 0.006 Da and 40 ppm, respectively. The second peptide search function was not performed. Other parameters were left as default. Unique spectral counts were derived from MaxQuant evidence.txt output by summing the MS/MS count values for evidence entries mapping to a single protein group, as determined by the Protein group IDs field. Evidence entries assigned to more than one protein group were excluded. Gel band fractions were pooled per condition by summing counts across raw files within each experiment. Identifications not meeting the FDR cut-off and contaminant proteins were removed from the data before further processing in Perseus version 1.5.6.0 (63). Common contaminants and non-specific binding proteins to GFP alone, under different experimental conditions, were removed from the final list of proteins identified by MS prior to further analysis. Gene Ontology Molecular Function analysis was performed using ShinyGO v0.85, using Ensembl release 113 and STRING-db v12 (64).

## Data Availability

All of the data associated with this study are included within the article or the supplemental material. Further information can be obtained from the corresponding author upon reasonable request.

## SUPPLEMENTAL MATERIAL

Supplemental material jvi.00582-26-s0001.pdf Figures S1 to S6.

ASM does not own the copyrights to Supplemental Material that may be linked to, or accessed through, an article. The authors have granted ASM a non-exclusive, world-wide license to publish the Supplemental Material files. Please contact the corresponding author directly for reuse.
