# Supplementary material for: The FACT complex associates with the RPA complex at viral replication compartments to promote adenovirus infection
Source: J Virol. 2026 May 26;100(6):e00582-26. doi: 10.1128/jvi.00582-26 (PMC13288986; doi:10.1128/jvi.00582-26)
Supplement: Supplemental material — Figures S1 to S6. [file jvi.00582-26-s0001.pdf]

Figure S1

A) RPA1+ Ad5

B) RPA1+ Ad12

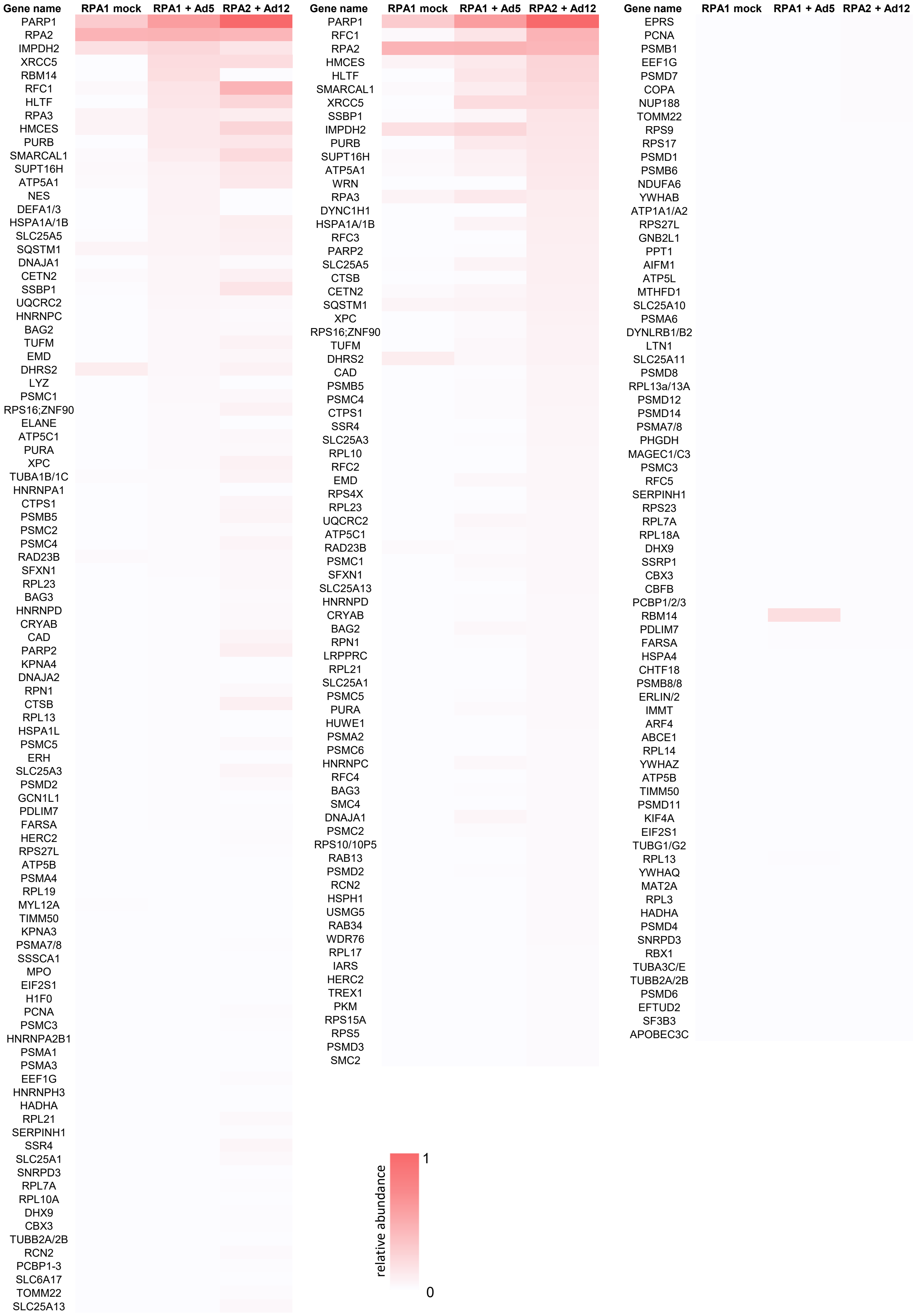

Figure S2

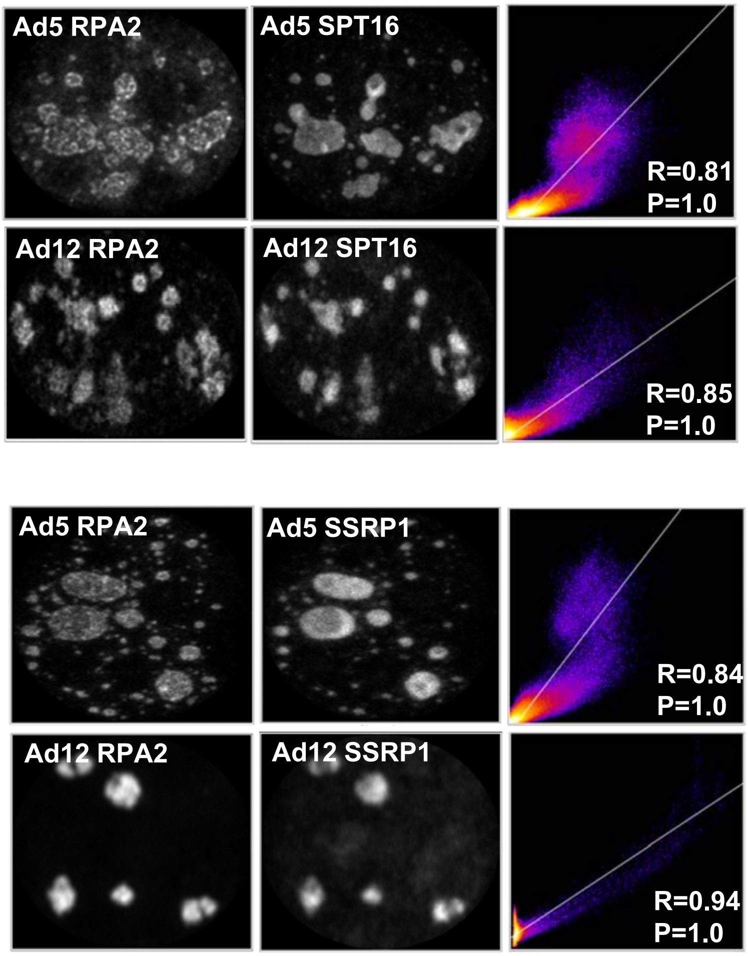

Ad5 RPA2
Ad5 SPT16
R=0.81
P=1.0
Ad12 RPA2
Ad12 SPT16
R=0.85
P=1.0
Ad5 RPA2
Ad5 SSRP1
R=0.84
P=1.0
Ad12 RPA2
Ad12 SSRP1
R=0.94
P=1.0

Figure S3

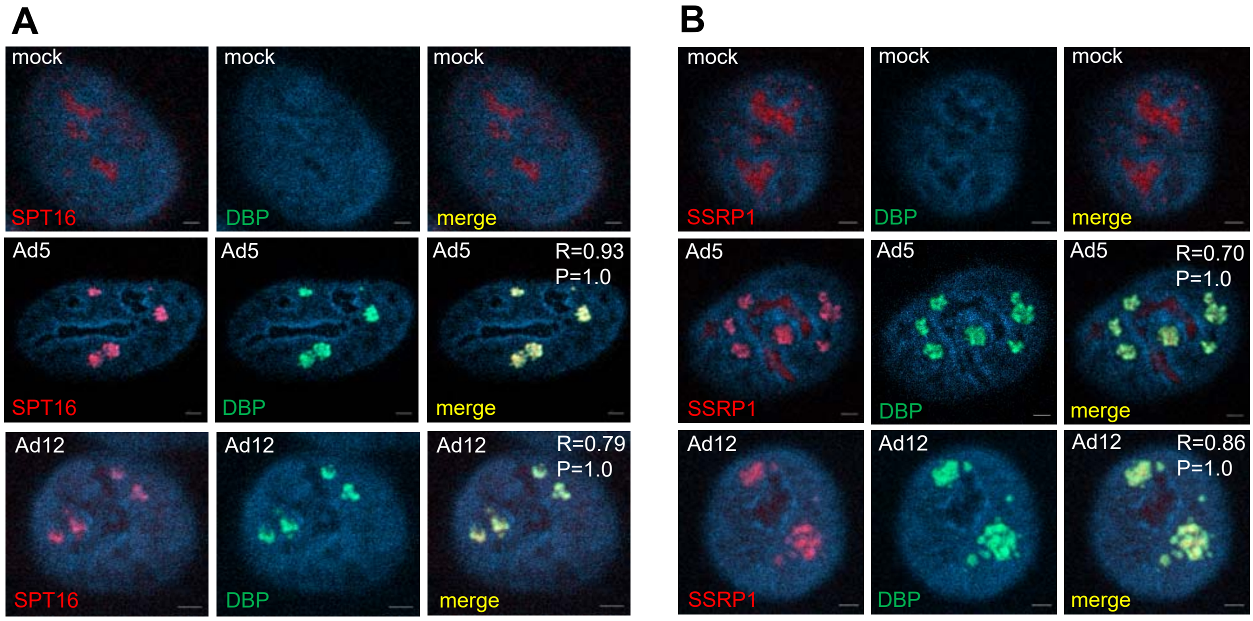
A
mock
SPT16
mock
DBP
mock
merge
Ad5
SPT16
Ad5
DBP
Ad5
R=0.93
P=1.0
merge
Ad12
SPT16
Ad12
DBP
Ad12
R=0.79
P=1.0
merge
B
mock
SSRP1
mock
DBP
mock
merge
Ad5
SSRP1
Ad5
DBP
Ad5
R=0.70
P=1.0
merge
Ad12
SSRP1
Ad12
DBP
Ad12
R=0.86
P=1.0
merge

Figure S4

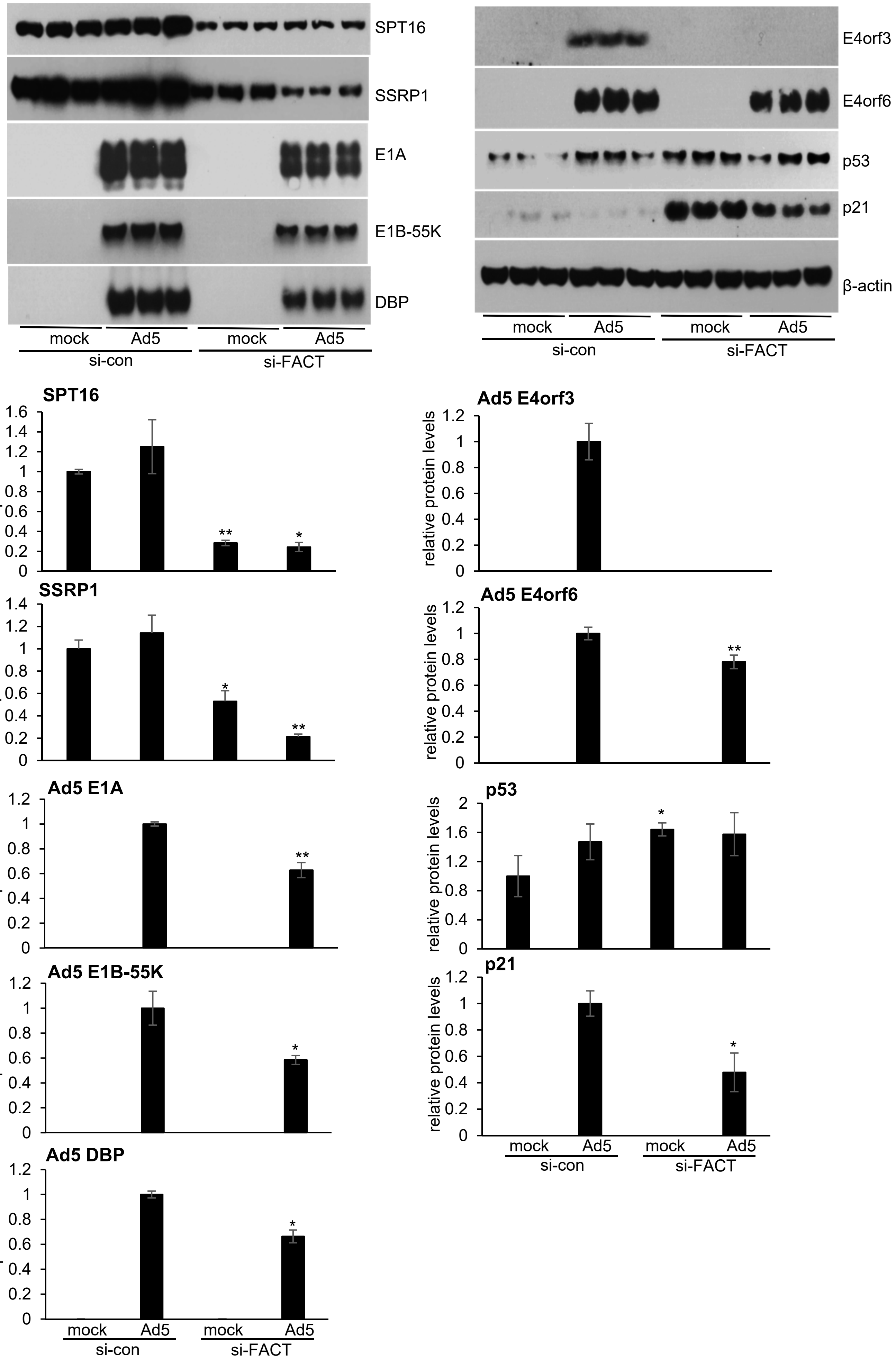

SPT16
SSRP1
E1A
E1B-55K
DBP
mock
Ad5
mock
Ad5
si-con
si-FACT
E4orf3
E4orf6
p53
p21
β-actin
mock
Ad5
mock
Ad5
si-con
si-FACT
SPT16
SSRP1
Ad5 E1A
Ad5 E1B-55K
Ad5 DBP
Ad5 E4orf3
Ad5 E4orf6
p53
p21
relative protein levels
mock
Ad5
mock
Ad5
si-con
si-FACT

Figure S5

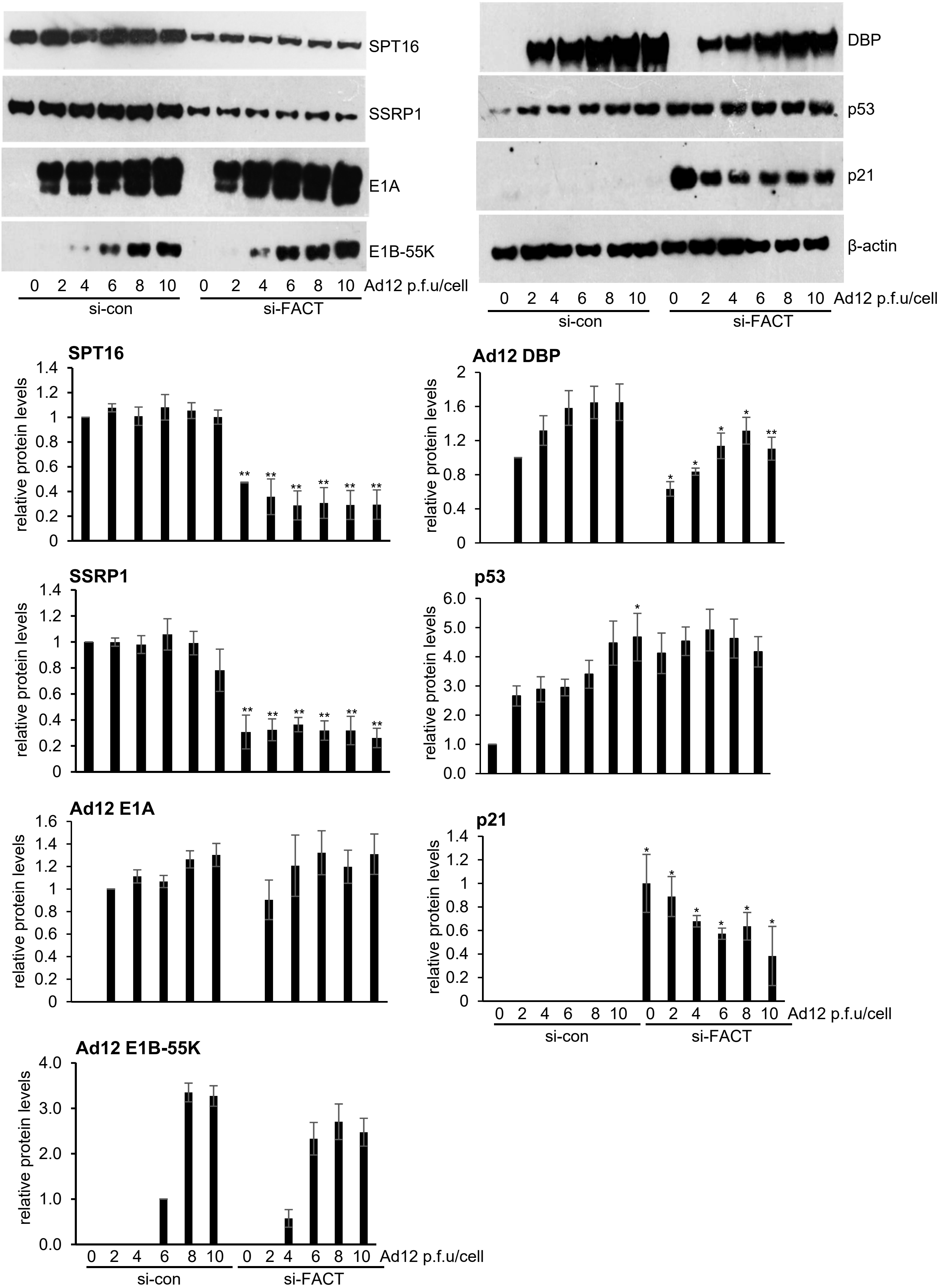

SPT16
SSRP1
E1A
E1B-55K
0 2 4 6 8 10 0 2 4 6 8 10 Ad12 p.f.u/cell
si-con
si-FACT
DBP
p53
p21
β-actin
0 2 4 6 8 10 0 2 4 6 8 10 Ad12 p.f.u/cell
si-con
si-FACT
SPT16
Ad12 DBP
SSRP1
p53
Ad12 E1A
p21
Ad12 E1B-55K
relative protein levels
0 2 4 6 8 10 0 2 4 6 8 10 Ad12 p.f.u/cell
si-con
si-FACT

Figure S6

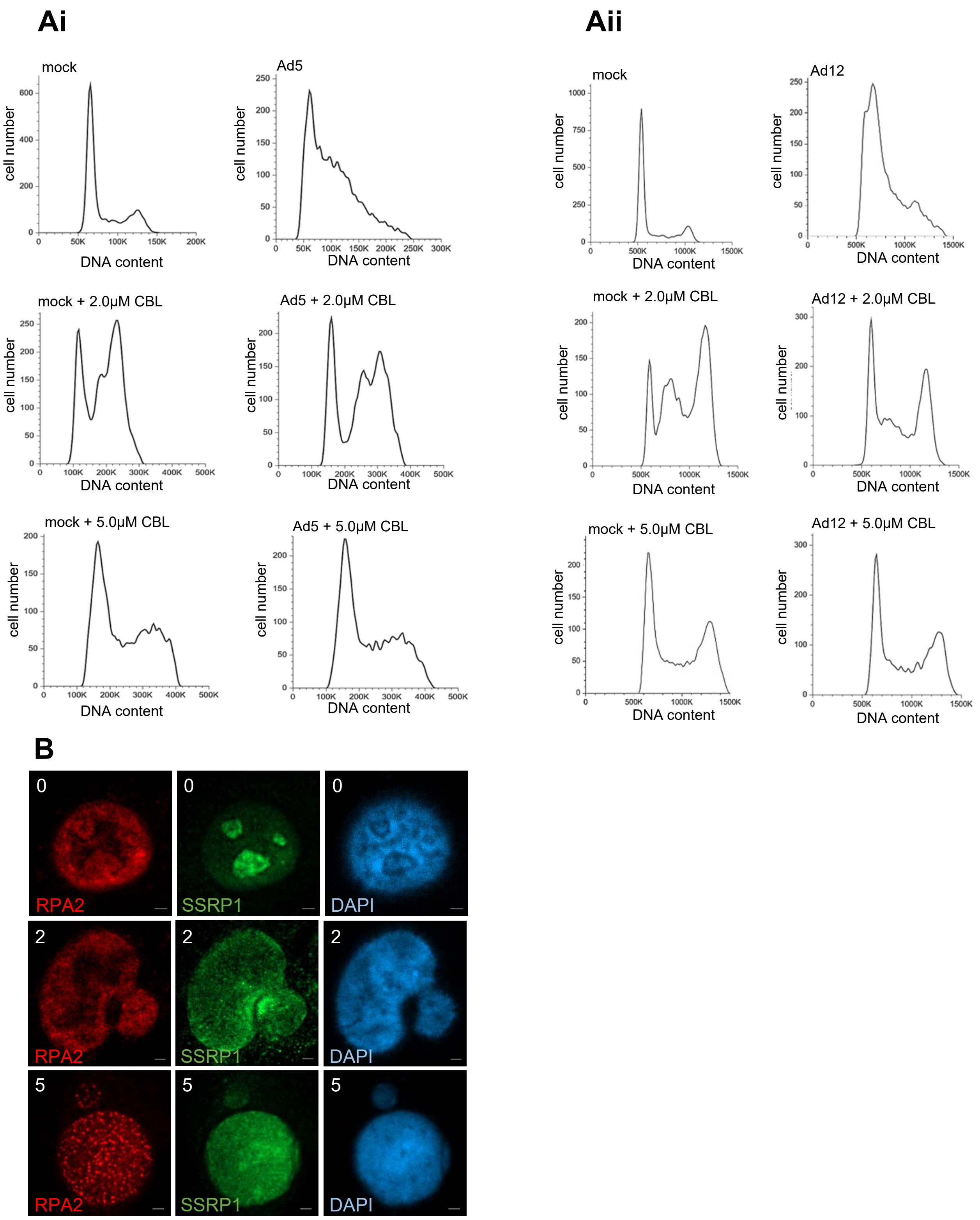

Ai
Aii
mock
Ad5
mock + 2.0μM CBL
Ad5 + 2.0μM CBL
mock + 5.0μM CBL
Ad5 + 5.0μM CBL
mock
Ad12
mock + 2.0μM CBL
Ad12 + 2.0μM CBL
mock + 5.0μM CBL
Ad12 + 5.0μM CBL
cell number
DNA content
B
0
2
5
RPA2
SSRP1
DAPI

Supplemental Figure legends

Figure S1. Heat maps showing relative abundance of all cellular proteins recruited to GFP-RPA1 during Ad5 and Ad12 infection. (A) Heat map showing relative abundance of all proteins identified by mass spectrometry that associated with GFP-RPA1 in Ad5-infected cells relative to mock-infected and Ad12-infected GFP-RPA1 U2OS cells. (B) Heat map showing relative abundance of all proteins identified by mass spectrometry that associated with GFP-RPA1 in Ad12-infected cells relative to mock-infected and Ad5-infected GFP-RPA1 U2OS cells. Relative abundance was calculated using total spectral counts for each protein identified.

Figure S2. Regions of interest (ROIs) from confocal images presented in Fig. 2 were selected for the determination of colocalization between RPA2 and SPT16, and RPA2 and SSRP1, in both Ad5- and Ad12- infected A549 cells. Colocalization was determined using Coloc2 in Fiji 2020-10. The Pearson's coefficient (R value) and the Costes P value are indicated in the 2D correlation intensity plot for the red channel (y-axis) v green channel (x-axis) for each image.

Figure S3. SPT16 and SSRP1 colocalize with DBP at VRCs in Ad5- and Ad12- infected cells. A549 cells were either mock-infected or infected with Ad5 or Ad12 at 10 pfu/cell. After 24 h, cells were fixed in methanol and co-stained with anti-Ad5 DBP, or anti-Ad12 DBP, and anti-SPT16 antibodies (A), or co-stained with anti-Ad5 DBP, or anti-Ad12 DBP, and anti-SSRP1 antibodies (B). Cells were counter-stained with the appropriate species-specific Alexa Fluor 488 and Alexa Fluor 594 antibodies and DAPI before visualisation with a Zeiss LSM880 confocal microscope. Scale bar = 2 μM. Colocalization for the ROIs indicated was determined using Coloc2 in Fiji 2020-10, whereby the Pearson's coefficient and the Costes P value were determined, as indicated in the merged image. ROIs displayed highly positive Pearson's correlation coefficients and significant Costes P values.

Figure S4. FACT component knockdown attenuates early gene product expression following Ad5 infection. A549 cells were treated with either non-silencing (si-con) or siRNA oligonucleotides specific to both SPT16 and SSRP1 (si-FACT). After 48 h, cells were infected with Ad5 at 10 pfu/cell for 18 h, after which cell lysates were prepared, separated by SDS-PAGE and analyzed by WB. Antibodies raised against E1A, E1B-55K, DBP, E4orf3, E4orf6 and β-actin were used to assess early viral protein levels. The relative (to β-actin) protein abundance ratio for early gene products was determined by densitometric analysis with Image J (n=3) and representative of similarly designed experiments. Data was analyzed using a two-tailed paired t-test. Bar chart values +/-S.D. indicate the relative expression levels. p-values: * $p \leq 0.05$, ** $p \leq 0.01$. Statistical differences were calculated between Ad-infected samples treated with non-silencing oligonucleotides v samples treated with SPT16 and SSRP1 oligonucleotides.

Figure S5. FACT component knockdown attenuates DBP expression following Ad12 infection. A549 cells were treated with either non-silencing (si-con) or siRNA oligonucleotides specific to both SPT16 and SSRP1 (si-FACT). After 48 h, cells were infected with Ad12 at 0, 2, 4, 6, 8 and 10 pfu/cell for 24 h, after which cell lysates were prepared, separated by SDS-PAGE and analyzed by WB. Antibodies raised against E1A, E1B-55K, DBP and β-actin were used to assess early viral protein levels. The relative (to β-actin) protein abundance ratio for early gene products was determined by densitometric analysis with Image J (n=3). Data was analyzed using a two-tailed paired t-test. Bar chart values +/-S.D. indicate the relative expression levels. p-values: * $p \leq 0.05$, ** $p \leq 0.01$. Statistical differences were calculated between Ad-infected samples treated with non-silencing oligonucleotides v samples treated with SPT16 and SSRP1 oligonucleotides.

Figure S6. FACT inhibitor CBL0137 promotes nuclear blebbing and aneuploidy at high doses. A549 cells were mock-infected or infected with Ad5 (Ai) or Ad12 (Aii) at 10 pfu/cell prior to incubation with different doses of CBL0137 (CBL). At 24 h post-Ad5 infection, and 32 h post-Ad12 infection, cells were fixed in 70% (v/v) ethanol, subjected to RNAse A treatment, resuspended in PBS containing propidium iodide and subjected to flow cytometry using a Beckman Coulter CytoFLEX S analyzer. Representative images shown in Ai and Aii were processed with Floreada.io. Images indicate anomalous S phase peak observed following treatment of mock-infected, Ad5-infected or Ad12-infected cells with 2.0 μM CBL0137, and to a lesser extent, with 5.0 μM CBL0137. (B) A549 cells were either mock-treated (0) or treated with with 2.0 μM CBL0137 (2), or 5.0 μM CBL0137 (5). After 24 h, cells were fixed in methanol and co-stained with anti-RPA2 and anti-SSRP1 antibodies. Cells were counter-stained with the appropriate species-specific Alexa Fluor 488 and Alexa Fluor 594 antibodies and DAPI before visualisation with a Zeiss LSM880 confocal microscope. Scale bar = 2 μM. Note the nuclear blebbing following 2.0 μM CBL0137 treatment and the RPA damage-like foci following 5.0 μM CBL0137 treatment.
